# Predictive value of baseline, cumulative exposure and annual mean changes in TyG-related indices for cardiovascular events in older rural adults with cardiovascular-kidney-metabolic syndrome stages 0–3: a cohort study

**DOI:** 10.3389/fendo.2026.1952253

**Published:** 2026-09-30

**Authors:** Yangzhen Xue, Qingxiu Tian, Rong Zhang, Jiayou Chu, Guilin Liu, Huishu Sun, Yong Qin, Xiaodan Yuan

**Affiliations:** 1 School of Nursing, Nanjing University of Chinese Medicine, Nanjing, Jiangsu, China; 2 Department of Nutrition, Xuyi County T.C.M. Hospital, Huai’an, Jiangsu, China; 3 Department of Anesthesiology, Xuyi County T.C.M. Hospital, Huai’an, Jiangsu, China; 4 Department of Public Health, Affiliated Hospital of Integrated Traditional Chinese and Western Medicine, Nanjing University of Chinese Medicine, Nanjing, Jiangsu, China

**Keywords:** basic public health service, cardiovascular disease, cardiovascular-kidney-metabolic(CKM) syndrome, insulin resistance, TyG-related indices

## Abstract

**Background:**

The triglyceride-glucose (TyG) index and its derivatives are cost-effective markers of insulin resistance and cardiovascular risk. However, the comparative predictive value of baseline levels, cumulative exposure, and annual mean changes in TyG-related indices remains unclear. This study aimed to systematically compare the predictive performance of multiple dimensions of TyG-related indices for cardiovascular events in rural older adults with cardiovascular-kidney-metabolic (CKM) syndrome stages 0–3.

**Methods:**

This cohort study enrolled 4,169 participants aged ≥65 years with no history of cardiovascular events. Longitudinal data from annual standardized health examinations under the National Basic Public Health Services Project were used to calculate baseline, cumulative exposure, and annual mean change values of TyG-related indices. Associations, dose-response relationships, and predictive performance were assessed via accelerated failure time models, restricted cubic spline analyses, time-dependent receiver operating characteristic analyses, and model fit statistics. Linear mixed-effects models were applied to delineate longitudinal trajectories of these indices. Sensitivity analyses were further conducted to verify the robustness of the main findings.

**Results:**

During 6 years of follow-up, 822 cardiovascular events were documented. Cardiovascular risk increased progressively with advancing CKM syndrome stages (*P* = 0.004). Baseline and cumulative TyG-WC and TyG-WHtR were consistently and strongly associated with incident cardiovascular events (all *P* < 0.001), with cumulative measures demonstrating slightly stronger associations. Dose-response analyses revealed a linear relationship between TyG and cardiovascular events, whereas TyG-BMI, TyG-WC, and TyG-WHtR exhibited nonlinear associations. In terms of association strength, most annual mean change measures were not significantly associated with outcomes, only TyG-BMI showed a weak association(*P* = 0.033) and was the only indicator with a statistically significant time-to-CVD interaction in longitudinal trajectory analysis. Nevertheless, all annual mean change indicators generally demonstrated poorer predictive performance than baseline and cumulative indicators for cardiovascular events. Among all indices, baseline and cumulative TyG-WC showed the optimal predictive performance across follow-up periods, and baseline TyG-WC had predictive utility comparable to cumulative TyG-WC. Sensitivity analyses further confirmed the robustness of these key findings.

**Conclusion:**

Baseline and cumulative TyG-related indices demonstrated stronger associations with cardiovascular events compared with annual mean changes, suggesting that sustained metabolic exposure is more clinically informative than short-term fluctuation. Baseline and cumulative TyG-WC demonstrated relatively better predictive performance and, especially baseline measure, may serve as a simple and low-cost auxiliary tool for cardiovascular risk stratification in resource-constrained primary care settings, though it should not replace conventional cardiovascular risk-assessment instruments.

## 1 Introduction

In 2023, the American Heart Association (AHA) put forward the term cardiovascular-kidney-metabolic (CKM) syndrome for the first time, defining it as a systemic disorder that stems from the interconnected pathophysiological processes linking obesity, diabetes, chronic kidney disease (CKD), and cardiovascular disease (CVD) (1). CKM syndrome has emerged as a major public health challenge. In China, the prevalence of CKM syndrome rose from 77.1% in 2010 to 83.7% in 2019, corresponding to an estimated affected population of 875 million individuals (2). This upward epidemiological trend coincides with the national population aging and persistent alterations in dietary patterns and lifestyle behaviors. The AHA has emphasized that a top public health priority is to prevent progression from the largely symptom-free preclinical stages of CKM syndrome stages 0–3 to advanced stage 4. Notably, subclinical cardiac and renal impairment has already initiated in individuals with CKM syndrome stages 0–3. Once patients advance to stage 4, they’re confronted with overt cardiovascular events, irreversible organ dysfunction, and an elevated risk of premature mortality (1, 3). Patients with cardiovascular disease often suffer from impaired health-related quality of life, attributable to prolonged therapeutic regimens, a spectrum of physical symptoms, and the financial burden imposed by medical expenses (4, 5).

Currently, the burden of cardiovascular events in China is markedly higher in rural areas compared with urban regions (719.38 vs. 550.60 per 100,000 population) (6). Moreover, there are disparities in public health development between urban and rural areas in China: primary medical resources in rural regions are relatively scarce, and the accessibility of public health services is lower than that in urban areas. To address this predicament and narrow the urban-rural health gap, the state has implemented inclusive and standardized public health services. Centered on inclusiveness, accessibility, and prevention, the Chinese National Basic Public Health Service Project currently comprises 12 categories of core services, among which “health management for the elderly” is intended to provide an annual health check-up for individuals aged 65 years and above. Given the importance of the preventive window and the special circumstances in rural settings, it is crucial to identify reliable screening markers for predicting the development of CVD in older rural adults with CKM syndrome stages 0–3 from the annual health check-up, thereby facilitating targeted disease prevention and timely clinical intervention.

Insulin resistance (IR) plays a central role in the pathogenesis of CKM syndrome by linking metabolic dysregulation with atherosclerotic progression (7).In recent years, the triglyceride-glucose (TyG) index has emerged as a practical surrogate marker of IR (8).Furthermore, composite indices combining TyG with obesity measures—such as triglyceride-glucose index-body mass index(TyG-BMI), triglyceride-glucose index-waist circumference index(TyG-WC), and triglyceride-glucose index-waist to-height ratio index(TyG-WHtR)—have been shown to exhibit superior predictive performance for cardiovascular outcomes compared with TyG index alone (9–11). However, most of the existing evidence on TyG-related indices for cardiovascular risk prediction is limited by the reliance on single baseline measurements and a focus on middle-aged cohorts (12–14).Notably, glycemic and lipid profiles are inherently dynamic and may fluctuate over time due to lifestyle modifications, pharmacological interventions, and the progression of comorbid conditions (15), meaning single baseline measurements cannot adequately capture the long-term cumulative metabolic burden driving cardiovascular risk. Although recent studies on CKM syndrome stages 0–3 have evaluated the cumulative exposure of TyG, there has been no systematic comparison of baseline, cumulative, or annual mean metrics for TyG-obesity composite indices (16, 17).The incremental predictive value of annual mean changes in these indices beyond baseline and cumulative exposure remains unclear.

We conducted a prospective cohort study using longitudinal health examination data from rural older adults based on the National Basic Public Health Service Project. The objectives were threefold: (1) to evaluate the associations of baseline levels, cumulative exposure, and annual mean changes in TyG-related indices with incident cardiovascular events; (2) to compare the predictive performance of these indices; and (3) to identify simple and scalable markers for cardiovascular risk stratification among older rural adults with CKM syndrome stages 0–3 in resource-limited primary care settings. Clarifying these relationships may enable more accurate cardiovascular risk stratification and inform targeted preventive strategies for this population.

## 2 Methods

### 2.1 Study population

This retrospective cohort study included elderly residents aged ≥65 years with CKM syndrome stages 0–3 at four primary healthcare centers in Huai’an City, Jiangsu Province, between 2019 and 2025. Participants were consecutively enrolled. Individuals were excluded if they met any of the following criteria: (1) incomplete baseline information, including missing data on medical history (e.g., diabetes, hypertension, cardiovascular disease, CKD) or lifestyle factors (smoking and drinking status); (2) missing measurements for key clinical variables, including fasting plasma glucose (FPG), triglycerides (TG), high-density lipoprotein cholesterol (HDL-C), low-density lipoprotein cholesterol (LDL-C), body mass index (BMI), waist circumference (WC), systolic blood pressure (SBP), or diastolic blood pressure (DBP); or (3) a documented history of cardiovascular events within two years prior to baseline. This study was approved by the Ethics Committee of Jiangsu Province Hospital on Integration of Chinese and Western Medicine (approval number: 2026-LWKYZ-022). The requirement for written informed consent was waived due to the retrospective nature of the study, in accordance with local ethical standards. The study adhered to the principles of the Declaration of Helsinki. A flowchart of participant selection and exclusion steps is depicted in **Figure 1**.

**Figure 1:**
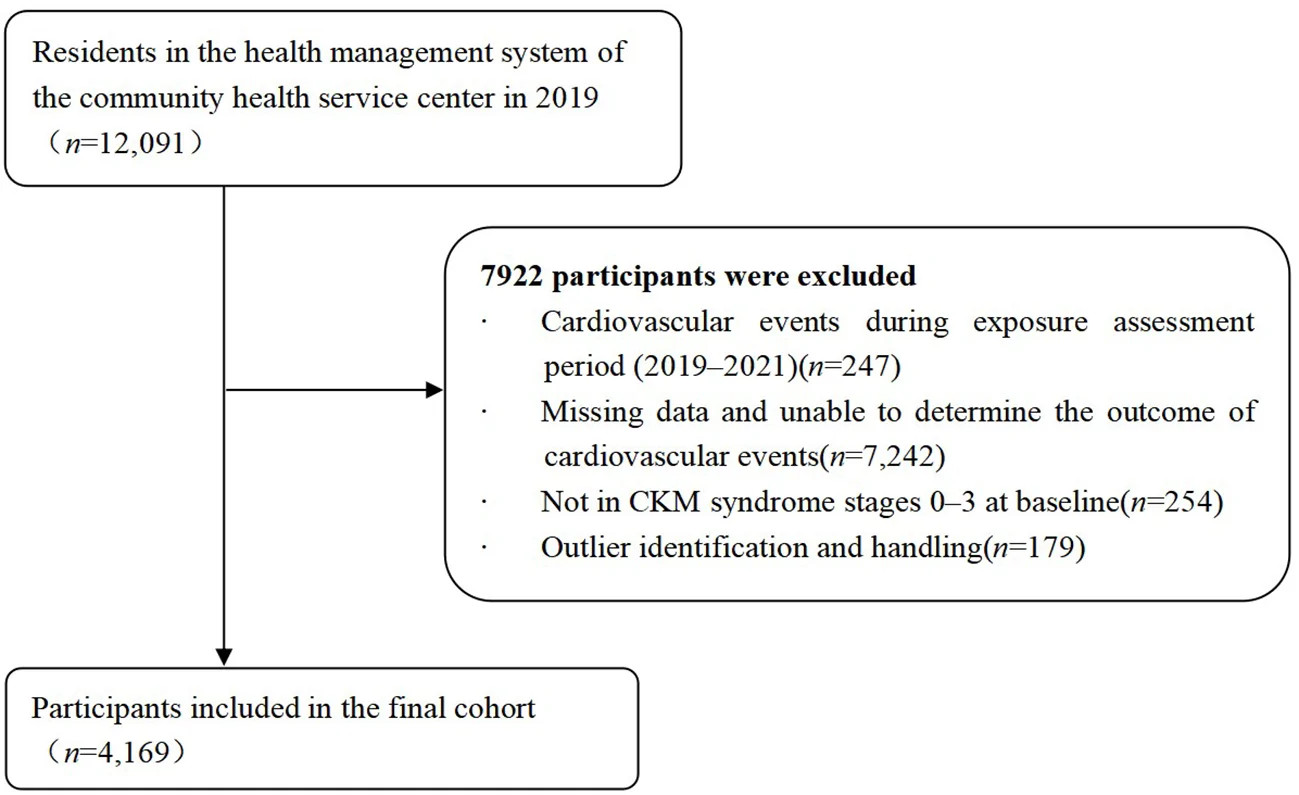
Flowchart of participants selection.

### 2.2 Variable definitions

All clinical data were extracted from participants’ medical records by trained clinicians using standardized procedures. Information collected included demographic characteristics, anthropometric measurements, blood pressure parameters, lifestyle factors (smoking and drinking status), and medical history, including hypertension, diabetes mellitus, myocardial infarction, stroke, and coronary revascularization. Laboratory measurements included FPG, total cholesterol (TC), TG, LDL-C, HDL-C, and creatinine (Cr). All blood samples were collected from peripheral veins in the early morning after an overnight fast of at least 8 hours. BMI was calculated as weight (kg) divided by height squared (m²). Waist to-height ratio (WHtR) was calculated as WC divided by height. Blood pressure was measured using a standard sphygmomanometer according to established guidelines, with participants resting for at least 5 minutes and refraining from smoking or caffeine intake for at least 30 minutes prior to measurement. The estimated glomerular filtration rate (eGFR) was calculated using the Chronic Kidney Disease Epidemiology Collaboration (CKD-EPI) equation. All measurements were conducted in accordance with standardized protocols to ensure data quality and consistency. Hypertension was defined as a self-reported physician diagnosis, current use of antihypertensive medications, or both (18). Diabetes mellitus was defined as a self-reported diagnosis, use of insulin or oral hypoglycemic agents, or fasting plasma glucose ≥7.0 mmol/L (19). Moderate-high risk CKD was defined as an eGFR of 30 to less than 60 mL/min per 1.73 m², and very high-risk CKD was defined as an eGFR below 30 mL/min per 1.73 m² (20).

### 2.3 Exposure assessment

Exposure variables included baseline measurements (2019), cumulative exposure, and annual mean changes in TyG-related indices during the exposure assessment period (2019–2021). The TyG-related indices were calculated as follows:


TyG=ln[TG(mg/dL)×FPG(mg/dL)/2]

$$\text{BMI}=\text{weight}(\text{kg})/{\text{height}}^{2}({\text{m}}^{2})$$

TyG−BMI=TyG×BMI

TyG−WC=TyG×WC

WHtR=WC(cm)/height(cm)

TyG−WHtR=TyG×WHtR

Cumulative exposure indices were calculated using repeated measurements collected between 2019 and 2021 to reflect long-term metabolic burden. This analytical approach captures sustained exposure levels, reduces regression dilution bias, and improves risk prediction in longitudinal cohort studies (21, 22). For each TyG-related index (TyG-X), cumulative exposure was computed as time-weighted averages across follow-up intervals. The 2-year and 3-year cumulative exposures were calculated based on measurements obtained in 2019–2020 and 2019–2021, respectively. The mean annual change in TyG-related indices was estimated using the trend slope over the 3-year period (2019–2021). The formulas for cumulative TyG-X and mean change in TyG-X were presented below:


$$2-year Cumulative TyG-X=\frac{{\text{TyG-X}}_{2019}{\text{+TyG-X}}_{2020}}{2}$$

$$3-year Cumulative TyG-X=\frac{{\text{TyG-X}}_{2019}{+2\times \text{TyG-X}}_{2020}{\text{+TyG-X}}_{2021}}{4}$$

$$3-year mean change=\frac{{\text{TyG-X}}_{2021}{\text{ - TyG-X}}_{2019}}{2}$$

Here, TyG-X_2019_, TyG-X_2020_, and TyG-X_2021_ refer to measurements from individual follow-up assessments. Time intervals were incorporated to account for uneven time gaps across examinations.

### 2.4 Staging classification of CKM syndrome (0–4)

According to the AHA Presidential Advisory on CKM Syndrome (1), Stage 0 indicates the absence of CKM-related risk factors. Stage 1 is characterized by excess or dysfunctional adiposity without metabolic abnormalities. Stage 2 includes metabolic diseases or moderate-to-high-risk CKD. Stage 3 represents subclinical CVDor a risk-equivalent pathway based on a high predicted 10-year CVD risk or very high-risk CKD. Specifically, the 10-year predicted risk was estimated with Predicting Risk of Cardiovascular Disease Events (PREVENT) model (23). Participants were classified as Stage 3 when their 10-year predicted risk ≥20%, or when they met criteria for very high-risk CKD. Stage 4 refers to the occurrence of clinical CVD, including coronary heart disease, heart failure, stroke, peripheral artery disease, or atrial fibrillation, and is further subdivided into stage 4a (without kidney failure) and stage 4b (with kidney failure). The stages of CKM syndrome were defined as shown in **Supplementary Table S1**.

### 2.5 Endpoints

The primary outcome of our study was the incidence of cardiovascular events. Endpoint events were documented when participants experienced any of the following conditions: heart failure, myocardial infarction, coronary revascularization, hemorrhagic stroke, ischemic stroke, or lacunar cerebral infarction (1). Events were identified based on physician diagnosis during follow-up. Cardiovascular mortality was excluded from the composite endpoint. Time-to-event was calculated from 2019 baseline to the first cardiovascular event or the end of follow-up, whichever came first. Event-free participants were censored at their last follow-up date.

### 2.6 Data cleaning and missing value handling

Longitudinal patterns of continuous variables were used to detect outliers. Observations with annual changes beyond ±2 standard deviations relative to the population mean were removed (n = 179). A total of 4,169 participants remained in the final sample after data cleaning. Multiple imputation by chained equations (MICE) was used to handle missing data (24). Two imputation methods—predictive mean matching (PMM) and classification and regression trees (CART)—were used to assess robustness. Five imputed datasets were generated for each method, and effect estimates were pooled using Rubin’s rules (25).

### 2.7 Statistical analysis

All statistical analyses were performed using R software (version 4.4.2). Normally distributed continuous variables were presented as mean ± standard deviation and compared between groups using the independent samples t-test. Non-normally distributed continuous variables were described as median and interquartile range [M(Q1, Q3)] and compared using the Mann-Whitney U test. Categorical variables were summarized as frequencies and percentages [n (%)], with the chi-square (χ²) test used to evaluate between-group differences. Kaplan-Meier survival analysis with the log-rank test was used to compare event-free survival across different CKM syndrome stages.

To investigate associations between TyG-related indices and cardiovascular events, the accelerated failure time (AFT) model, a parametric survival regression method that directly quantifies the relationship between exposure variables and time-to-event outcomes (26, 27), was applied as the primary analytical approach. The semi-parametric Cox proportional hazards model, by contrast, requires the proportional hazards(PH) assumption, i.e., constant hazard ratios throughout follow-up (28). We formally evaluated this assumption using Schoenfeld residuals, which indicated violation of the PH assumption for several TyG-related indices. Under PH violation, Cox-derived hazard ratios represent time-averaged effects and fail to reliably summarize the exposure-outcome association over time. In contrast, the AFT framework does not rely on the PH assumption and yields more interpretable and efficient effect estimates (29, 30). Among the candidate distributions, the log-normal distribution was selected based on the Akaike information criterion (AIC), which balances model fit against complexity. The AFT model with log-normal distribution was therefore adopted as the primary model (**Supplementary Tables S2**–**S4**). The AFT model generates time ratios (TRs), which represent the multiplicative change in survival time associated with a one-unit increase in the exposure and therefore indicate whether a higher TyG-related index shortens or prolongs the time to cardiovascular events (30). Specifically, a time ratio (TR) of 1.0 indicates no association between the index and cardiovascular events; a TR < 1.0 suggests that higher index levels shorten the time to event; and a TR > 1.0 indicates that higher levels are associated with longer event-free time. Regression coefficients from the AFT model were exponentiated to obtain TRs. For each TyG-related index, TRs and corresponding 95% CIs were calculated per one standard deviation (SD) increment and per interquartile range (IQR) increment across quartiles (Q2–Q4 vs. Q1, where Q4 denotes the highest and Q1 the lowest category).Three sequentially adjusted models were constructed to control for confounding. Model 1 was unadjusted. Model 2 was adjusted for age, sex, smoking status, and alcohol consumption. Model 3 was further adjusted for hypertension history, diabetes history, systolic blood pressure, and LDL-C. Multicollinearity was assessed using the variance inflation factor (VIF); all VIF values were less than 5, indicating no severe multicollinearity. Based on Model 3, restricted cubic splines (RCS) with four pre-specified knots were used to explore potential non-linear relationships between baseline TyG-related indices and cardiovascular outcomes.

Longitudinal trajectories of TyG-related indices from 2019 to 2021 were visualized using line graphs. Linear mixed-effects models (LMM) were applied to assess whether temporal changes differed between participants with and without subsequent cardiovascular events. Models included fixed effects for time (treated as a continuous variable), event status (event vs. non-event), and their interaction (time × event status), with a significant interaction term indicated divergent temporal trends between groups. A random intercept was specified for each participant to account for within-individual correlation of repeated measures. All statistical analyses were conducted across five imputed datasets, and pooled interaction p-values and marginal means ± standard errors (SE) were derived using Rubin’s rules.

Time-dependent receiver operating characteristic (TD-ROC) curves and Harrell’s C-statistic were used to assess the discriminatory power of each index for cardiovascular events prediction, with the area under the curve (AUC) and C-index as the main metrics. The time-dependent AUC (TD-AUC) was calculated at 4-year, 5-year, and 6-year time points. Predictive performance and its stability over time were evaluated at these follow-up time points. A heatmap was constructed to visually summarize predictive performance, with darker colors representing higher AUC values and better discriminative ability. Bootstrap resampling was conducted for pairwise comparisons of AUCs. The Benjamini-Hochberg (BH) procedure was applied to correct for multiple comparisons, control the false discovery rate (FDR), and avoid Type I error inflation. To assess whether adding TyG-related indices improved predictive performance, model fit was compared before and after incorporating these variables using AIC, Bayesian information criterion (BIC), and the likelihood ratio test (LRT). To verify the robustness of the main results derived from PMM multiple imputation, sensitivity analyses were performed to address potential biases related to missing data handling and sample selection. Two sensitivity strategies were used: (1) multiple imputation using CART algorithm as an alternative missing data approach; (2) complete-case analysis using the original unimputed dataset.

## 3 Results

### 3.1 Participants characteristics

**Table 1** presents the baseline characteristics of the 4,169 rural older adults included in the final analysis. The median age was 69 years, and women accounted for 53.7%. Regarding CKM syndrome distribution, stage 0–1 comprised 18.5% of participants, stage 2 accounted for 33.8%, and stage 3 was the most prevalent group at 47.7%. The CVD group tended to be older and had a higher burden of hypertension and diabetes. Additionally, BMI, WC, WHtR, baseline and cumulative TyG-BMI, baseline and cumulative TyG-WC, baseline and cumulative TyG-WHtR, and mean change TyG-BMI were all higher in the CVD group (all *P* < 0.05). The two groups showed no significant differences in gender, smoking or drinking status, SBP, FPG, Cr, TG, LDL-C, baseline TyG, mean change TyG, mean change TyG-WC, or mean TyG-WHtR (all *P* > 0.05).

**Table 1:** Baseline characteristics of study participants.

Characteristics	Overall (n=4,169)	No CVD (n=3,347)	CVD (n=822)	*P* value
Age, years	69.00 (66.00, 74.00)	69.00 (66.00, 74.00)	70.00 (67.00, 74.00)	<0.001
Gender, n (%)				0.162
Male	1930 (46.3%);	1565 (46.8%);	365 (44.4%);	
Female	2239 (53.7%)	1782 (53.2%)	457 (55.6%)	
Smoking status, n (%)				0.995
Non-smokers	3564 (85.5%)	2864 (85.6%)	700 (85.2%)	
Smokers	605 (14.5%)	483 (14.4%)	122 (14.8%)	
Drinking status, n (%)				0.999
Never	3817 (91.6%)	3066 (91.6%)	751 (91.4%)	
Ever	352 (8.4%)	281 (8.4%)	71 (8.6%)	
Hypertension, n (%)				<0.001
No	2892 (69.4%)	2387 (71.3%)	505 (61.4%)	
Yes	1277 (30.6%)	960 (28.7%)	317 (38.6%)	
Diabetes, n (%)				<0.001
No	3815 (91.5%)	3095 (92.5%)	720 (87.6%)	
Yes	354 (8.5%)	252 (7.5%)	102 (12.4%)	
SBP, mmHg	134 (128, 144)	134 (128, 144)	132 (128, 145)	0.862
DBP, mmHg	82 (78, 88)	82 (78, 88)	81 (76, 88)	<0.001
BMI, kg/m²	25.08 (23.06, 27.30)	25.06 (23.01, 27.23)	25.23 (23.34, 27.64)	<0.001
FPG, mg/dL	95.4 (86.4, 108)	95.4 (86.4, 108)	95.4 (86.4, 108)	0.998
Cr, mg/dL	0.87 (0.73, 1.07)	0.87 (0.73, 1.05)	0.86 (0.72, 1.11)	0.385
eGFR, mL·min^-1^·1.73m^-2^	76.43 (56.41, 89.98)	76.72 (57.12, 90.28)	75.84 (54.50, 88.87)	0.040
TC, mg/dL	181.42 (159.03, 206.90)	184.12 (160.58, 206.90)	176.79 (154.01,206.90)	<0.001
TG, mg/dL	110.63 (84.96, 150.45)	109.74 (84.96, 147.80)	112.40 (84.96, 157.53)	0.283
LDL-C, mg/dL	91.10 (78.74, 108.85)	91.10 (79.13, 108.08)	92.64 (76.43, 111.17)	0.933
HDL-C, mg/dL	52.50 (45.55, 63.30)	52.50 (45.16, 63.30)	53.27 (46.32, 63.69)	0.023
WC, cm	85.00 (80.00, 90.00)	85.00 (80.00, 90.00)	86.00 (80.00, 93.00)	<0.001
WHtR	0.53 (0.50, 0.57)	0.53 (0.50, 0.57)	0.54 (0.50, 0.58)	<0.001
Baseline TyG	8.59 (8.28, 8.93)	8.58 (8.28, 8.93)	8.61 (8.30, 8.98)	0.260
Baseline TyG-BMI	215.80 (194.82, 239.45)	215.27 (194.77, 238.77)	217.61 (195.50, 241.70)	<0.001
Baseline TyG-WC	728.26 (671.10, 795.47)	725.15 (669.75, 790.43)	743.07 (674.57, 816.34)	<0.001
Baseline TyG-WHtR	4.58 (4.19, 5.02)	4.56 (4.19, 4.98)	4.70 (4.23, 5.16)	<0.001
2-year cumulative TyG	8.67 (8.43, 8.94)	8.66 (8.43, 8.93)	8.71 (8.45, 8.99)	<0.001
2-year cumulative TyG-BMI	217.68 (198.83, 239.59)	216.92 (198.13, 238.70)	220.62 (200.98, 244.00)	<0.001
2-year cumulative TyG-WC	734.62 (683.70, 791.02)	731.11 (682.14, 785.82)	750.15 (697.09, 810.79)	<0.001
2-year cumulative TyG-WHtR	4.61 (4.27, 5.00)	4.58 (4.26, 4.97)	4.73 (4.32, 5.10)	<0.001
3-year cumulative TyG	8.72 (8.49, 8.97)	8.71 (8.49, 8.96)	8.76 (8.51, 9.03)	<0.001
3-year cumulative TyG-BMI	218.76 (200.48, 240.98)	217.94 (199.77, 240.01)	223.31 (203.17, 244.55)	<0.001
3-year cumulative TyG-WC	737.70 (690.37, 790.94)	734.30 (688.83, 785.49)	753.12 (701.61, 811.95)	<0.001
3-year cumulative TyG-WHtR	4.62 (4.32, 5.00)	4.61 (4.30, 4.98)	4.73 (4.39, 5.09)	<0.001
3-year mean change TyG	0.09 (-0.11, 0.30)	0.09 (-0.11, 0.30)	0.09 (-0.10, 0.29)	0.685
3-year mean change TyG-BMI	2.24 (-5.86, 10.39)	2.09 (-6.16, 10.47)	2.90 (-4.79, 10.10)	0.005
3-year mean change TyG-WC	5.26 (-21.63, 33.59)	5.61 (-21.28, 33.21)	3.11 (-22.37, 33.32)	0.758
3-year mean change TyG-WHtR	0.03 (-0.13, 0.21)	0.04 (-0.13, 0.21)	0.02 (-0.14, 0.21)	0.736
CKM syndrome stages				0.148
CKM 0-1	771 (18.5)	635 (19)	136 (16.5)	
CKM 2	1410 (33.8)	1132 (33.8)	278 (33.8)	
CKM 3	1988 (47.7)	1580 (47.2)	408 (49.6)	

Data imputed with predictive mean matching (PMM); values were expressed as median (interquartile range) or number (percentage) as appropriate. The following abbreviations were used: SBP, systolic blood pressure; DBP, diastolic blood pressure; BMI, body mass index; FPG, fasting plasma glucose; Cr, creatinine; TC, total cholesterol; TG, triglycerides; LDL-C, low-density lipoprotein cholesterol; HDL-C, high-density lipoprotein cholesterol; WC, waist circumference; WHtR, waist-to-height ratio; TyG, triglyceride glucose index; TyG-BMI, triglyceride-glucose index-body mass index; TyG-WC, triglyceride-glucose index-waist circumference index; Triglyceride-glucose index-waist-to-height ratio index; CKM, cardiovascular-kidney-metabolic syndrome.

### 3.2 Cumulative incidence of cardiovascular events by CKM syndrome stages 0–3

**Figure 2** displays the Kaplan-Meier curves for cardiovascular events among the rural older adults with CKM syndrome stages 0–3. The curves demonstrated that cardiovascular event incidence differed significantly across CKM syndrome stages (*P* = 0.004). Furthermore, with advancing CKM syndrome stages, the cumulative incidence of cardiovascular events exhibited a progressive, stepwise increase.

**Figure 2:**
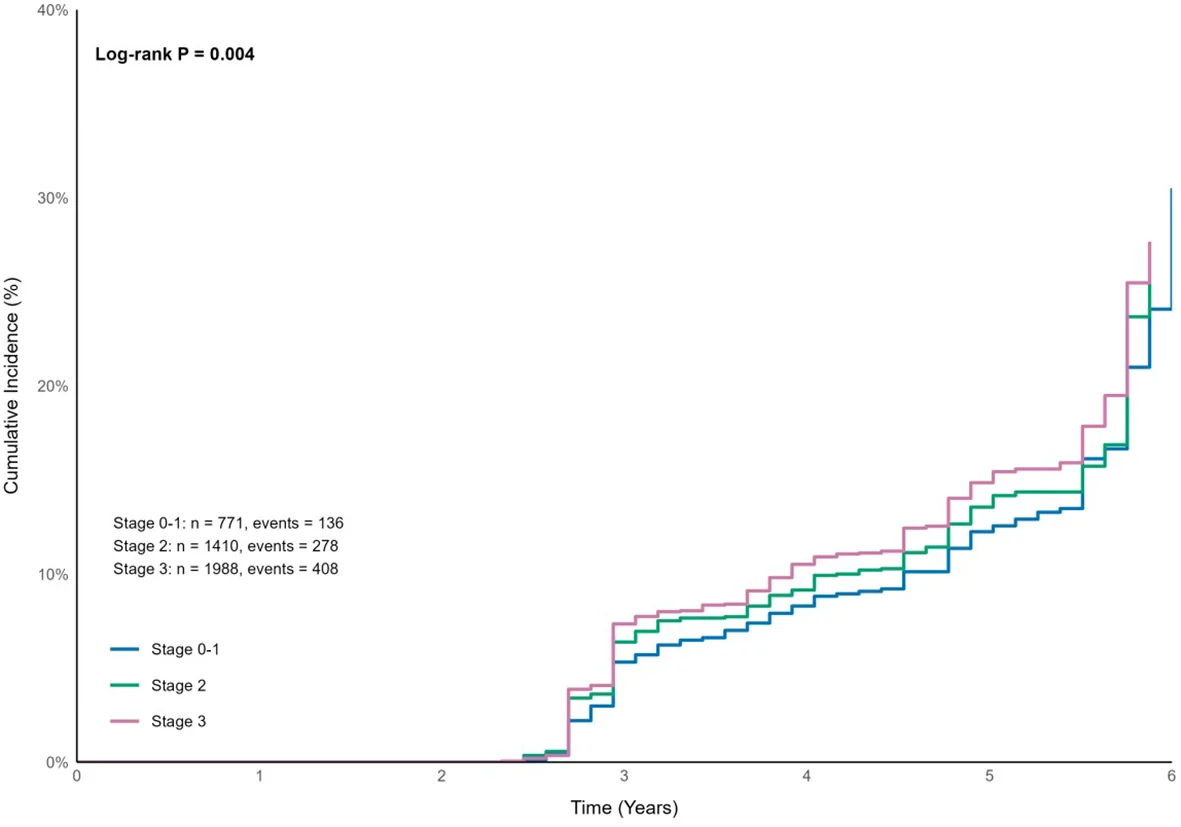
Kaplan-Meier curves for cardiovascular events cumulative incidence by CKM syndrome stages 0-3(data imputed with PMM). CKM, cardiovascular-kidney-metabolic syndrome; PMM, predictive mean matching; Stage 0-1 (blue); Stage 2 (green); Stage 3 (purple-red). Log-rank test: P = 0.004.

### 3.3 Associations of TyG-related indices (baseline, cumulative and mean change) with cardiovascular events

As presented in **Table 2**, AFT model analyses demonstrated that TyG-WC and TyG-WHtR were significantly associated with shorter time to cardiovascular events onset, even after full adjustment for covariates in Model 3. In comparison, TyG and TyG-BMI exhibited relatively weak and inconsistent associations with cardiovascular outcomes.

**Table 2:** Time ratios of associations between baseline, cumulative and annual mean change TyG-related indices with cardiovascular events among rural older adults with CKM syndrome stages 0–3 (data imputed with PMM).

Exposures	Model 1		Model 2		Model 3	
	TR (95%CI)	*P* value	TR (95%CI)	*P* value	TR (95%CI)	*P* value
Baseline TyG						
Per SD	0.994 (0.969,1.020)	0.674	0.995 (0.970,1.021)	0.709	0.996 (0.970,1.023)	0.794
Q1	Reference		Reference		Reference	
Q2	0.997 (0.924,1.076)	0.933	0.991 (0.919,1.069)	0.818	0.990 (0.919,1.067)	0.795
Q3	1.000 (0.929,1.076)	0.997	1.000 (0.930,1.075)	0.998	0.994 (0.924,1.069)	0.874
Q4	0.965 (0.898,1.037)	0.333	0.966 (0.899,1.037)	0.337	0.968 (0.899,1.041)	0.379
*P* for trend	0.367		0.404		0.413	
Baseline TyG-BMI						
Per SD	0.975 (0.950,1.000)	0.049	0.977 (0.952,1.002)	0.076	0.982 (0.956,1.008)	0.172
Q1	Reference		Reference		Reference	
Q2	1.038 (0.964,1.118)	0.321	1.034 (0.960,1.114)	0.372	1.035 (0.961,1.115)	0.361
Q3	1.006 (0.936,1.082)	0.869	1.005 (0.935,1.080)	0.890	1.014 (0.942,1.091)	0.716
Q4	0.973 (0.905,1.046)	0.452	0.979 (0.910,1.053)	0.573	0.991 (0.920,1.069)	0.823
*P* for trend	0.292		0.397		0.634	
Baseline TyG-WC						
Per SD	0.945 (0.921,0.970)	<0.001	0.944 (0.920,0.969)	<0.001	0.945 (0.920,0.971)	<0.001
Q1	Reference		Reference		Reference	
Q2	1.057 (0.978,1.143)	0.161	1.045 (0.967,1.130)	0.262	1.047 (0.969,1.132)	0.241
Q3	0.989 (0.915,1.068)	0.771	0.983 (0.909,1.062)	0.662	0.982 (0.908,1.063)	0.657
Q4	0.884 (0.821,0.952)	0.001	0.882 (0.819,0.950)	0.001	0.887 (0.822,0.958)	0.002
*P* for trend	<0.001		<0.001		<0.001	
Baseline TyG-WHtR						
Per SD	0.947 (0.923,0.972)	<0.001	0.952 (0.927,0.978)	<0.001	0.953 (0.927,0.981)	<0.001
Q1	Reference		Reference		Reference	
Q2	1.003 (0.927,1.085)	0.939	1.002 (0.926,1.083)	0.969	1.002 (0.927,1.084)	0.959
Q3	0.972 (0.900,1.050)	0.471	0.974 (0.900,1.053)	0.499	0.973 (0.899,1.054)	0.502
Q4	0.857 (0.798,0.921)	<0.001	0.868 (0.806,0.934)	<0.001	0.871 (0.807,0.940)	<0.001
*P* for trend	<0.001		<0.001		<0.001	
2-year cumulative TyG						
Per SD	0.979 (0.954,1.004)	0.099	0.980 (0.955,1.005)	0.120	0.984 (0.958,1.011)	0.239
Q1	Reference		Reference		Reference	
Q2	1.006 (0.931,1.087)	0.887	1.006 (0.932,1.086)	0.881	1.007 (0.934,1.087)	0.849
Q3	0.985 (0.916,1.059)	0.686	0.987 (0.918,1.061)	0.717	0.986 (0.917,1.060)	0.697
Q4	0.922 (0.858,0.990)	0.026	0.926 (0.862,0.994)	0.034	0.930 (0.864,1.002)	0.056
*P* for trend	0.018		0.025		0.041	
2-year cumulative TyG-BMI						
Per SD	0.964 (0.940,0.989)	0.005	0.965 (0.941,0.990)	0.007	0.972 (0.947,0.998)	0.034
Q1	Reference		Reference		Reference	
Q2	0.961 (0.890,1.038)	0.310	0.958 (0.887,1.033)	0.264	0.965 (0.894,1.041)	0.352
Q3	0.962 (0.891,1.039)	0.322	0.960 (0.889,1.036)	0.288	0.971 (0.899,1.048)	0.448
Q4	0.910 (0.843,0.982)	0.015	0.913 (0.846,0.986)	0.020	0.929 (0.859,1.005)	0.067
*P* for trend	0.015		0.021		0.077	
2-year cumulative TyG-WC						
Per SD	0.935 (0.912,0.960)	<0.001	0.934 (0.911,0.959)	<0.001	0.939 (0.914,0.964)	<0.001
Q1	Reference		Reference		Reference	
Q2	1.000 (0.925,1.080)	0.995	0.990 (0.917,1.069)	0.800	0.993 (0.919,1.072)	0.850
Q3	0.929 (0.863,1.000)	0.049	0.926 (0.861,0.996)	0.039	0.930 (0.864,1.001)	0.054
Q4	0.844 (0.786,0.907)	<0.001	0.841 (0.783,0.903)	<0.001	0.851 (0.791,0.915)	<0.001
*P* for trend	<0.001		<0.001		<0.001	
2-year cumulative TyG-WHtR						
Per SD	0.938 (0.915,0.963)	<0.001	0.943 (0.918,0.968)	<0.001	0.947 (0.921,0.974)	<0.001
Q1	Reference		Reference		Reference	
Q2	1.011 (0.930,1.098)	0.803	1.013 (0.933,1.101)	0.755	1.013 (0.932,1.100)	0.759
Q3	0.907 (0.840,0.980)	0.014	0.912 (0.844,0.985)	0.020	0.918 (0.848,0.993)	0.032
Q4	0.864 (0.803,0.930)	<0.001	0.876 (0.812,0.945)	0.001	0.887 (0.820,0.958)	0.002
*P* for trend	<0.001		<0.001		<0.001	
3-year cumulative TyG						
Per SD	0.975 (0.950,1.000)	0.046	0.976 (0.951,1.001)	0.060	0.982 (0.957,1.008)	0.173
Q1	Reference		Reference		Reference	
Q2	1.018 (0.945,1.097)	0.633	1.021 (0.948,1.099)	0.584	1.021 (0.949,1.099)	0.577
Q3	0.953 (0.883,1.029)	0.219	0.957 (0.887,1.034)	0.264	0.957 (0.887,1.033)	0.261
Q4	0.920 (0.856,0.989)	0.023	0.923 (0.858,0.993)	0.031	0.931 (0.864,1.003)	0.060
*P* for trend	0.005		0.007		0.017	
3-year cumulative TyG-BMI						
Per SD	0.958 (0.934,0.982)	0.001	0.958 (0.934,0.983)	0.001	0.965 (0.941,0.991)	0.008
Q1	Reference		Reference		Reference	
Q2	0.991 (0.915,1.073)	0.821	0.988 (0.914,1.069)	0.768	0.993 (0.918,1.074)	0.864
Q3	0.920 (0.855,0.989)	0.024	0.917 (0.853,0.986)	0.019	0.929 (0.863,0.999)	0.046
Q4	0.894 (0.831,0.963)	0.003	0.896 (0.832,0.966)	0.004	0.912 (0.846,0.984)	0.017
*P* for trend	<0.001		0.001		0.004	
3-year cumulative TyG-WC						
Per SD	0.932 (0.909,0.956)	<0.001	0.931 (0.908,0.955)	<0.001	0.937 (0.913,0.961)	<0.001
Q1	Reference		Reference		Reference	
Q2	1.010 (0.935,1.090)	0.808	1.003 (0.930,1.082)	0.946	1.003 (0.930,1.082)	0.936
Q3	0.929 (0.860,1.002)	0.058	0.924 (0.856,0.996)	0.040	0.932 (0.863,1.005)	0.068
Q4	0.842 (0.784,0.905)	<0.001	0.839 (0.781,0.901)	<0.001	0.851 (0.791,0.915)	<0.001
*P* for trend	<0.001		<0.001		<0.001	
3-year cumulative TyG-WHtR						
Per SD	0.936 (0.912,0.960)	<0.001	0.939 (0.915,0.965)	<0.001	0.945 (0.920,0.972)	<0.001
Q1	Reference		Reference		Reference	
Q2	0.954 (0.879,1.036)	0.263	0.955 (0.879,1.036)	0.265	0.960 (0.885,1.042)	0.328
Q3	0.883 (0.818,0.953)	0.001	0.888 (0.823,0.958)	0.002	0.894 (0.828,0.966)	0.004
Q4	0.840 (0.780,0.904)	<0.001	0.849 (0.787,0.917)	<0.001	0.863 (0.799,0.933)	<0.001
*P* for trend	<0.001		<0.001		<0.001	
3-year mean change TyG						
Per SD	0.991 (0.966,1.017)	0.513	0.992 (0.967,1.018)	0.549	0.996 (0.970,1.022)	0.768
Q1	Reference		Reference		Reference	
Q2	0.972 (0.902,1.047)	0.454	0.973 (0.904,1.047)	0.467	0.979 (0.910,1.053)	0.568
Q3	0.951 (0.879,1.029)	0.210	0.953 (0.880,1.032)	0.232	0.959 (0.887,1.038)	0.300
Q4	0.992 (0.921,1.068)	0.823	0.992 (0.922,1.068)	0.838	1.002 (0.930,1.079)	0.962
P for trend	0.712		0.724		0.805	
3-year mean change TyG-BMI						
Per SD	0.971 (0.945,0.998)	0.037	0.968 (0.942,0.995)	0.020	0.971 (0.944,0.998)	0.033
Q1	Reference		Reference		Reference	
Q2	0.931 (0.861,1.006)	0.070	0.930 (0.861,1.005)	0.067	0.936 (0.867,1.011)	0.092
Q3	0.891 (0.825,0.963)	0.004	0.888 (0.823,0.959)	0.003	0.897 (0.832,0.967)	0.005
Q4	0.948 (0.878,1.025)	0.179	0.941 (0.872,1.016)	0.122	0.948 (0.877,1.025)	0.179
*P* for trend	0.080		0.047		0.076	
3-year mean change TyG-WC						
Per SD	0.999 (0.973,1.026)	0.938	0.998 (0.971,1.025)	0.871	0.999 (0.972,1.026)	0.944
Q1	Reference		Reference		Reference	
Q2	0.994 (0.920,1.073)	0.869	0.991 (0.919,1.069)	0.816	0.996 (0.924,1.073)	0.913
Q3	1.057 (0.978,1.142)	0.163	1.050 (0.972,1.133)	0.213	1.053 (0.976,1.137)	0.182
Q4	1.015 (0.941,1.094)	0.707	1.012 (0.938,1.092)	0.754	1.015 (0.941,1.094)	0.701
P for trend	0.389		0.448		0.408	
3-year mean change TyG-WHtR						
Per SD	0.999 (0.973,1.026)	0.968	0.999 (0.972,1.025)	0.914	1.000 (0.973,1.027)	0.984
Q1	Reference		Reference		Reference	
Q2	0.995 (0.921,1.074)	0.895	0.990 (0.918,1.068)	0.799	0.996 (0.924,1.073)	0.914
Q3	1.053 (0.974,1.138)	0.195	1.043 (0.966,1.127)	0.280	1.048 (0.970,1.131)	0.235
Q4	1.018 (0.945,1.097)	0.634	1.017 (0.943,1.096)	0.668	1.019 (0.945,1.099)	0.620
*P* for trend	0.360		0.401		0.373	

PMM, predictive mean matching; TR, time ratio; CI, confidence interval; SD, standard deviations. Model 1 was unadjusted; Model 2 was adjusted for age, gender, smoking status and drinking status; Model 3 was adjusted for age, gender, smoking status, drinking status, SBP, diabetes, hypertension and LDL-C.

For baseline measurements, TyG-WC and TyG-WHtR remained independently associated with earlier cardiovascular events onset in the fully adjusted model. Per SD increment in baseline TyG-WC was associated with a 5.5% reduction in time to cardiovascular events (TR = 0.945, 95%CI 0.920–0.971, *P* < 0.001). A similar magnitude of association was observed for baseline TyG-WHtR, where in per SD increase corresponded to a 4.7% reduction in time to event (TR = 0.953, 95%CI 0.927–0.981, *P* < 0.001). In quartile stratified analyses, participants in Q4 exhibited the strongest association. Compared with Q1, participants in Q4 of baseline TyG-WC demonstrated an 11.3% reduction in time to cardiovascular events (TR = 0.887, 95%CI 0.822–0.958, *P* = 0.002), whereas those in Q4 of baseline TyG-WHtR showed a 12.9% reduction (TR = 0.871, 95%CI 0.807–0.940, *P* < 0.001). Significant linear trends across quartiles were observed for both indices (*P* for trend < 0.001). By contrast, no significant associations were observed for baseline TyG (TR = 0.996, *P* = 0.794) or TyG-BMI (TR = 0.982, *P* = 0.172) after full adjustment.

For cumulative exposure indices, the associations with cardiovascular events were generally stronger than those observed for single baseline measurements. Each SD increment in 2-year cumulative TyG-WC was associated with a 6.1% reduction in time to events (TR = 0.939, 95%CI 0.914–0.964, *P* < 0.001), with a nearly identical effect size observed for 3-year cumulative TyG-WC (TR = 0.937, 95%CI 0.913–0.961, *P* < 0.001). TyG-WHtR demonstrated a consistent dose–response pattern: each SD increase in 2-year cumulative exposure corresponded to a 5.3% reduction in time to cardiovascular events (TR = 0.947, 95%CI 0.921–0.974, *P* < 0.001), with a marginally stronger effect for 3-year cumulative exposure (TR = 0.945, 95%CI 0.920–0.972, *P* < 0.001). Both 2-year and 3-year cumulative TyG-BMI reached statistical significance after full adjustment (TR = 0.972, *P* = 0.034; TR = 0.965, *P* = 0.008, respectively), although with markedly smaller effect sizes compared with TyG-WC and TyG-WHtR. In contrast, cumulative TyG showed no significant association with cardiovascular events (2-year: TR = 0.984, *P* = 0.239; 3-year: TR = 0.982, *P* = 0.173).In quartile analyses of cumulative indices, participants in Q4 of 3-year cumulative TyG-WC had a 14.9% reduction in time to events (TR = 0.851, 95%CI 0.791–0.915, *P* < 0.001), while those in Q4 of 3-year cumulative TyG-WHtR had a 13.7% reduction (TR = 0.863, 95%CI 0.799–0.933, *P* < 0.001). Significant linear trends across quartiles were observed for both indices (*P* for trend < 0.001).

For annual mean change indices of TyG-related metrics, overall associations with cardiovascular events onset were weak and non-significant. Only the annual mean change TyG-BMI was associated with earlier cardiovascular events in the fully adjusted model: per SD increment was associated with a 2.9% reduction in time to events (TR = 0.971, 95%CI 0.944–0.998, *P* = 0.033). In quartile analyses, participants in Q3 of annual mean change in TyG-BMI showed a 10.3% reduction in time to cardiovascular events (TR = 0.897, 95%CI 0.832–0.967, *P* = 0.005). No significant associations were observed for annual mean changes in TyG, TyG-WC, or TyG-WHtR (all *P* > 0.05).

### 3.4 Dose-response relationships

We further evaluated the dose–response relationships between baseline TyG-related indices and cardiovascular events using RCS analyses, which are visually presented in **Figure 3**. After adjustment for confounding variables, baseline TyG demonstrated a linear association with cardiovascular events (P-nonlinearity = 0.369). However, markedly different patterns were observed for baseline TyG-BMI, TyG-WC, and TyG-WHtR. All three indices exhibited non-linear associations with cardiovascular events (all P*-*nonlinearity < 0.05). These non-linear patterns suggest that the association between these indices and cardiovascular risk was not strictly linear, with the risk increasing more steeply at higher levels. The estimated inflection points were 216.42 for TyG-BMI, 728.66 for TyG-WC, and 4.58 for TyG-WHtR.

**Figure 3:**
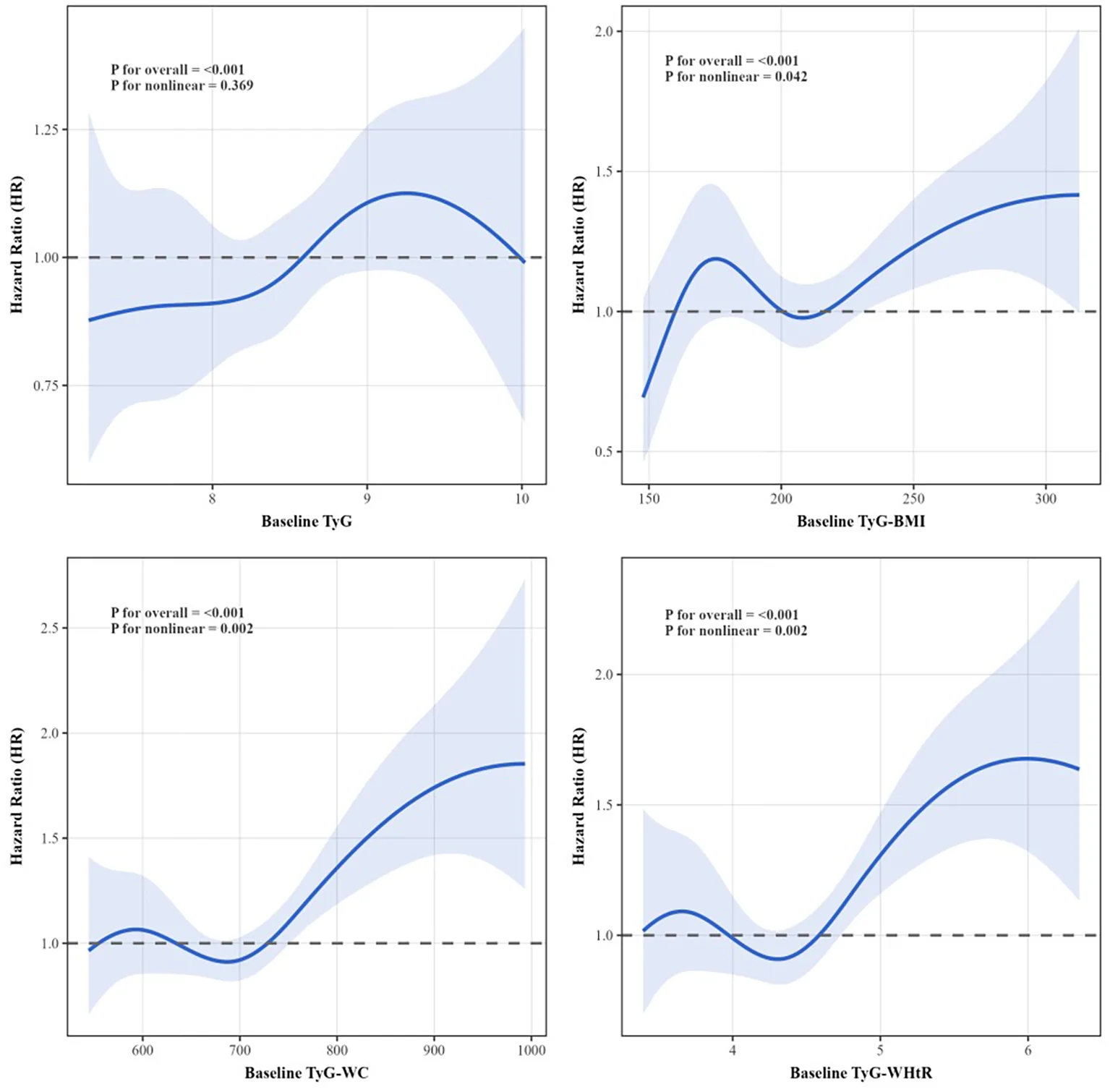
Restricted cubic spline analysis between baseline TyG-related indices and cardiovascular events in populations with CKM syndrome stages 0-3(data imputed with PMM). PMM, predictive mean matching; other abbreviations as in **Table 1**.

### 3.5 Predictive analysis

#### 3.5.1 Predictive performance

**Figure 4** presents the C-statistics for TyG-related indices according to CKM syndrome stages. In the overall cohort, the base model incorporating conventional covariates yielded a C-statistic of 0.591. Addition of baseline and cumulative TyG-WC increased the C-statistic to 0.598–0.602; the highest value (0.602) was observed for 3-year cumulative TyG-WC, followed by 2-year cumulative TyG-WC (0.601) and baseline TyG-WC (0.598). Baseline TyG, baseline TyG-BMI, and all mean change indices demonstrated marginal improvements in discrimination (C-statistics <0.595). The improvement in discrimination was most pronounced in early CKM syndrome stages. Throughout the entire follow-up period, predictive performance was better in CKM stages 0–1 than in stages 2–3, with higher C-statistics observed across all model specifications (all adjusted *P* < 0.05), supporting better predictive value of TyG-derived indices in early CKM stages. In stages 0–1, the base model incorporating conventional covariates yielded a C-statistic of 0.607. Addition of 3-year cumulative TyG-WC increased this to 0.623; baseline and 2-year cumulative TyG-WC yielded values of 0.622 and 0.621, respectively. In stages 2 and 3, the incremental gains were smaller, yet TyG-WC still outperformed other indices. Cumulative measures consistently surpassed baseline and mean change measures across all stages. In contrast, mean change metrics produced C-statistics comparable to the base model incorporating conventional covariates regardless of stage, and generally demonstrated poorer predictive performance than baseline and cumulative indicators.

**Figure 4:**
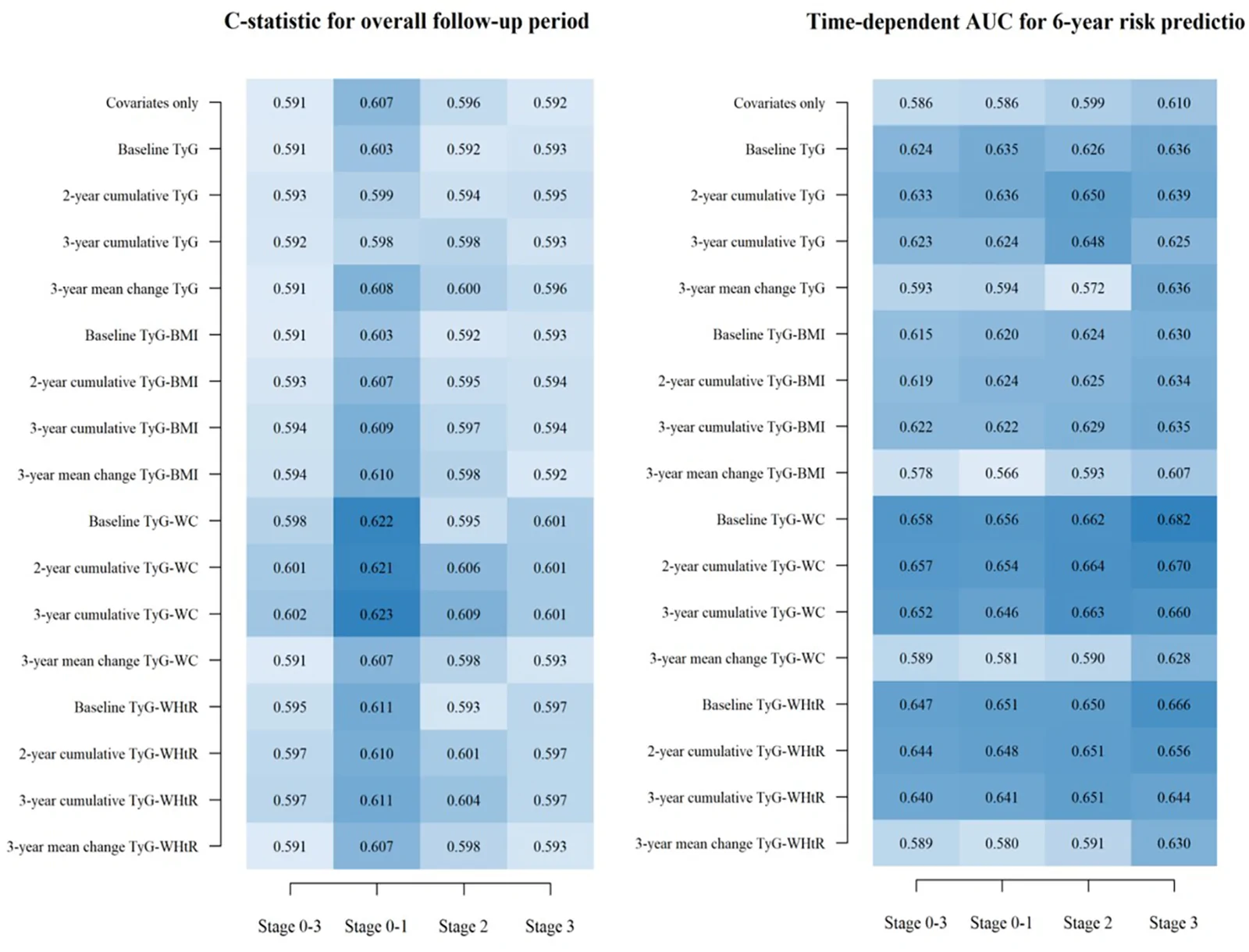
Predictive performance of TyG-related indices for cardiovascular events in populations with CKM syndrome stages 0-3(data imputed with PMM). AUC, area under the curve; PMM, predictive mean matching; other abbreviations as in **Table 1**.

TD-AUC for 6-year risk prediction demonstrated similar patterns but with higher absolute values. In the overall cohort, baseline TyG-WC (0.658) and 2-year cumulative TyG-WC (0.657) achieved the highest AUC values, compared with 0.586 for the base model incorporating conventional covariates. In stage 3, baseline TyG-WC (0.682) exhibited the strongest moderate discriminatory performance. In stages 0–1 and 2, cumulative TyG-WC maintained robust moderate discrimination, outperforming TyG, TyG-BMI, and mean change measures. Across all stages, baseline or cumulative TyG-WC consistently surpassed other indices, whereas mean change metrics provided minimal additional predictive value beyond the base model.

TD-AUC for 4-year and 5-year risk prediction showed patterns consistent with the 6-year results (**Supplementary Figure S5**). Across all CKM syndrome stages, baseline and cumulative TyG-WC consistently outperformed TyG, TyG-BMI, and 3-year mean change metrics, with the highest AUC values observed in CKM stages 0–1. Notably, baseline TyG-WC achieved the highest 4-year AUC (0.642) and 5-year AUC (0.641) in stages 0–1, while cumulative TyG-WC maintained robust moderate predictive performance across all follow-up periods. Mean change metrics showed marginal improvements over the base model incorporating conventional covariates, with limited incremental predictive value.

#### 3.5.2 Model comparison and incremental predictive value

Compared with the base model incorporating traditional cardiovascular risk factors, inclusion of baseline or cumulative TyG-related indices improved predictive performance (all adjusted *P* < 0.05) (**Supplementary Tables S10**–**S12**). Baseline TyG and baseline TyG-BMI models demonstrated comparable discrimination (adjusted *P* = 0.172) (**Supplementary Table S10**). Similarly, cumulative TyG and cumulative TyG-BMI models did not differ significantly across follow-up periods (2-year: adjusted *P* = 0.141; 3-year: adjusted *P* = 0.915) (**Supplementary Tables S11**, **S12**). However, models incorporating TyG-WC or TyG-WHtR consistently outperformed those including TyG or TyG-BMI, although some pairwise comparisons did not reach statistical significance, including TyG-WC vs. TyG (*P* = 0.087) and TyG-WHtR vs. TyG (*P* = 0.315) in 2-year cumulative models, as well as TyG-WHtR vs. TyG (*P* = 0.149) in 3-year cumulative models (**Supplementary Tables S11**, **S12**). Notably, TyG-WC exhibited marginally higher predictive performance relative to TyG-WHtR across all evaluation periods (baseline: ΔAUC = 0.011, *P* = 0.026; 2-year cumulative: ΔAUC = 0.013, *P* = 0.037; 3-year cumulative: ΔAUC = 0.012, *P* = 0.044) (**Supplementary Tables S10**–**S12**). Collectively, these findings indicated that TyG-WC may represent the optimal predictive indicator among the TyG-related indices evaluated.

Furthermore, models incorporating annual mean changes in TyG-related indices demonstrated no significant improvement over the base model (all adjusted *P* > 0.05), with no significant differences across mean change metrics (**Supplementary Table S13**). Moreover, all mean change models performed significantly worse than their baseline and cumulative counterparts (all adjusted *P* < 0.05) (**Supplementary Tables S14**–**S17**). For the TyG, the 2-year cumulative measure outperformed the 3-year cumulative measure (ΔAUC = 0.010, adjusted *P* = 0.008) (**Supplementary Table S14**), whereas for TyG-BMI, TyG-WC, and TyG-WHtR, baseline and cumulative assessments exhibited equivalent predictive utility (all adjusted *P* > 0.05) (**Supplementary Tables S15**–**S17**).

Longitudinal AUC comparisons across 48, 60, and 72 months revealed no significant differences for any model (all *P* > 0.05), indicating consistent predictive performance of TyG-related indices across follow-up durations (**Supplementary Table S26**).

LRT analysis confirmed that incorporation of cumulative TyG-WC or cumulative TyG-WHtR into the base model significantly enhanced model fit (both *P* < 0.001) (**Table 3**). Corresponding reductions in AIC and BIC further supported improved predictive performance after accounting for model complexity. Notably, inclusion of 3-year cumulative TyG-WC yielded the most substantial improvement (LRT χ² = 38.25, *P* < 0.001; AIC: 12764.60 vs. 12800.60; BIC: 12807.00 vs. 12838.30).

**Table 3:** Comparison and Incremental Predictive Value of cardiovascular events prediction model(data imputed with PMM).

Model variables	C-index	95%CI	Δ AIC	Δ BIC	LRT P-value
base model(basic covariates)	0.591	0.495–0.688	0.0	0.0	Ref
Basic + 3-year cumulative TyG-WC	0.602	0.506–0.698	-34.0	-29.3	<0.001
Basic + 2-year cumulative TyG-WC	0.601	0.505–0.697	-31.8	-27.1	<0.001
Basic + basline TyG-WC	0.598	0.502–0.694	-25.2	-20.5	<0.001
Basic + 3-year cumulative TyG-WHtR	0.597	0.501–0.693	-22.2	-17.5	<0.001
Basic + 2-year cumulative TyG-WHtR	0.597	0.500–0.693	-20.9	-16.2	<0.001
Basic + basline TyG-WHtR	0.595	0.499–0.691	-16.7	-12.0	<0.001
Basic + 3-year cumulative TyG-BMI	0.594	0.498–0.690	-9.5	-4.8	<0.001
Basic + 2-year cumulative TyG-BMI	0.593	0.496–0.689	-6.2	-1.5	0.003
Basic + 2-year cumulative TyG	0.593	0.496–0.689	-4.3	0.4	0.015
Basic + 3-year cumulative TyG	0.592	0.496–0.689	-4.3	0.4	0.015
Basic + basline TyG-BMI	0.591	0.495–0.687	-3.2	1.5	0.022
Basic + 3-year mean change TyG-BMI	0.594	0.498–0.690	-1.8	2.9	0.077
Basic + basline TyG	0.591	0.494–0.687	-1.1	3.6	0.087
Basic + 3-year mean change TyG	0.591	0.495–0.687	1.8	6.5	0.645
Basic + 3-year mean change TyG-WHtR	0.591	0.495–0.688	1.9	6.6	0.677
Basic + 3-year mean change TyG-WC	0.591	0.495–0.688	2.0	6.7	0.702

PMM, predictive mean matching; ΔAIC, difference in AIC compared to base model; ΔBIC, difference in BIC compared to base model; LRT, likelihood ratio test; base model include age, gender, smoking status, drinking status, SBP, diabetes, hypertension and LDL-C; other abbreviations as in **Table 1**.

### 3.6 Longitudinal trajectories of TyG-related indices through exposure period by CVD status

Longitudinal trajectories of TyG-related indices are shown in **Figure 5**. All indices exhibited an overall increasing trend over the 3-year follow-up period in both groups, with higher levels observed in participants who developed cardiovascular events compared with those who remained event-free. A statistically significant interaction between time and CVD status was identified exclusively for TyG-BMI (*P* = 0.026), indicating divergent temporal patterns between groups. In contrast, no significant time-by-CVD interactions were observed for TyG, TyG-WC, or TyG-WHtR (all *P* > 0.05). Although absolute levels remained consistently elevated in the CVD group, the magnitude of temporal fluctuation was insufficient to generate materially divergent trajectories between groups. These findings were consistent with the AFT analysis results, demonstrating that only the annual mean change in TyG-BMI was associated with cardiovascular risk.

**Figure 5:**
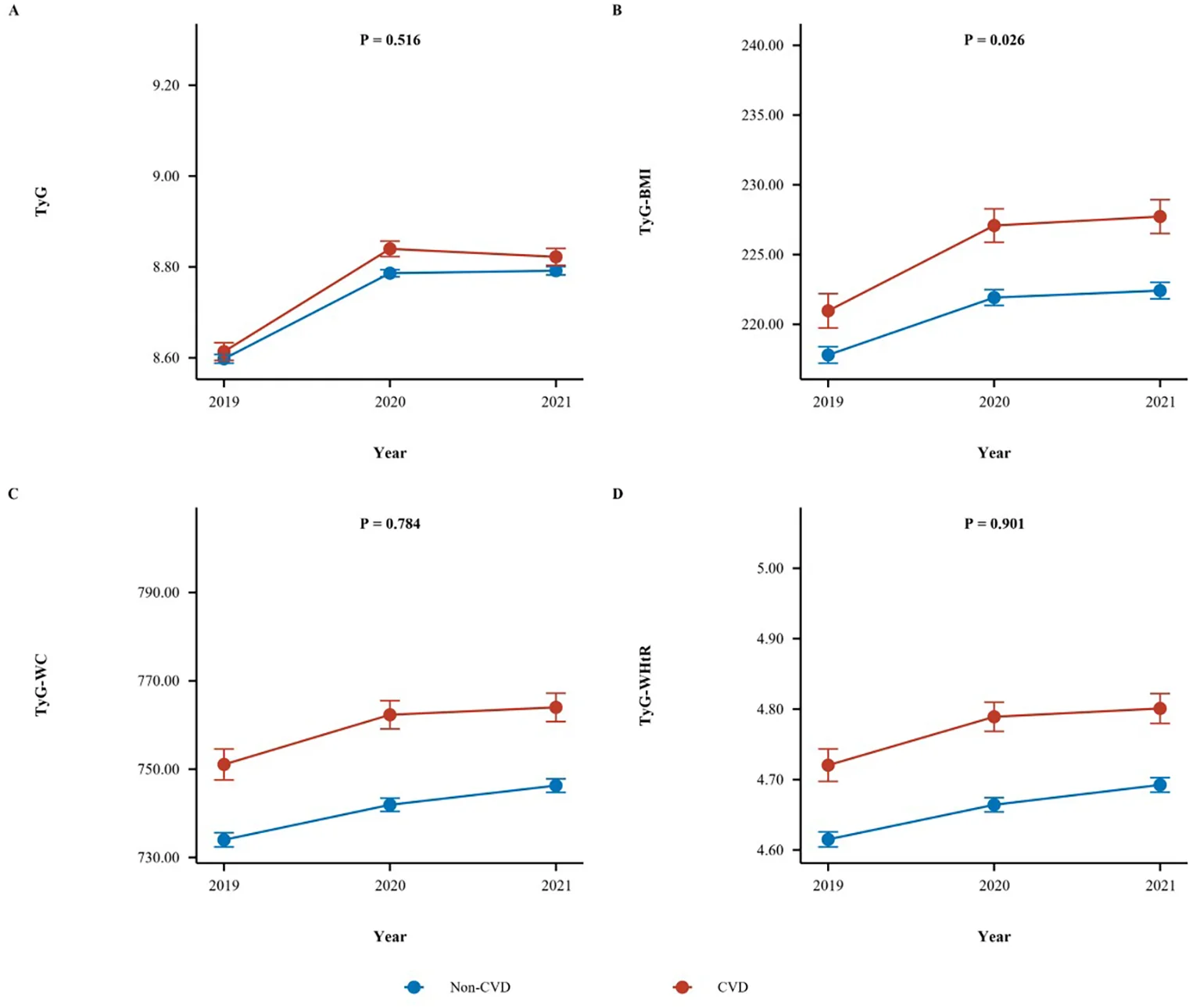
Longitudinal trajectories of TyG-related indices in populations with CKM syndrome stages 0-3 stratified by CVD status(data imputed with PMM). PMM, predictive mean matching; other abbreviations as in **Table 1**; data are presented as pooled mean values with standard error (SE) bars. The P-values represent the interaction between time and CVD status.

### 3.7 Sensitivity analysis

Kaplan-Meier survival analyses based on CART multiple imputation and complete case analysis were highly consistent with the primary analysis, confirming a stepwise increase in the cumulative incidence of cardiovascular events with higher CKM syndrome stage. The log-rank test was significant in the CART-imputed analysis (*P* = 0.004) and showed marginal significance in the complete-case analysis (*P* = 0.070), **Supplementary Figures S1** and **S2**. AFT models demonstrated that, after full adjustment, baseline and cumulative TyG-WC and TyG-WHtR remained robust independent predictors of earlier cardiovascular events, whereas TyG and annual mean change metrics were not statistically significant, and the association for TyG-BMI was unstable, mostly marginal associations (**Supplementary Tables S5**, **S6**). RCS sensitivity analyses further confirmed that the association patterns between various TyG-related indices and cardiovascular events were consistent with the primary analysis (**Supplementary Figures S3**, **S4**). Longitudinal trajectories of TyG-related indices through the exposure period by CVD status with CART-imputed dataset showed similar results relative to primary analysis (**Supplementary Figure S8**). C statistics and TD-AUC results for different models using the CART-imputed dataset were consistent with the primary findings (**Supplementary Figures S6**, **S7**). Predictive performances of original non-imputed data for cardiovascular events prediction were presented in **Supplementary Table S7**. In terms of prediction model fit, baseline and cumulative TyG-WC and TyG-WHtR significantly improved model fit (all LRT *P* < 0.05); among them, the 3-year cumulative TyG-WC (C-index 0.601, LRT *P* < 0.001) and the 2-year cumulative TyG-WHtR (C-index 0.612, LRT *P* = 0.007) showed relatively better performance in the two sensitivity analyses, respectively. In contrast, all annual mean change metrics did not improve model discrimination (**Supplementary Tables S8**, **S9**). The AUC comparison results for different models using the CART-imputed dataset were consistent with the primary findings (**Supplementary Tables S18**–**S25**, **S27**). Collectively, the CART multiple imputation and complete case analyses confirmed the stability of the primary findings. Baseline and cumulative TyG-WC and TyG-WHtR showed the stronger associations with incident CVD among all indices examined, although their overall predictive performance remained moderate. TyG-WC, in particular, exhibited similar predictive utility at baseline and cumulatively. These conclusions were consistent across different missing data methods and sample selection strategies.

## 4 Discussion

This study demonstrated that among rural older adults with CKM syndrome stages 0–3, the risk of cardiovascular events increased incrementally with advancing CKM syndrome stage. Cumulative TyG-BMI, baseline and cumulative TyG-WC, as well as baseline and cumulative TyG-WHtR, were all independent risk factors of cardiovascular events, with TyG-WC and TyG-WHtR exhibiting stronger associations. Dose-response analyses revealed a linear relationship between TyG and cardiovascular events, while TyG-BMI, TyG-WC, and TyG-WHtR each exhibited nonlinear associations with cardiovascular events. For predictive modeling, addition of baseline or cumulative TyG-related indices to the base model comprising traditional cardiovascular risk factors improved model goodness of fit, and inclusion of 3-year cumulative TyG-WC in particular enhanced model discrimination. AUC comparisons further confirmed that incorporation of baseline or cumulative TyG-WC and TyG-WHtR improved model discrimination, with baseline and cumulative TyG-WC demonstrating moderate incremental predictive value.

The utility of combined IR-adiposity indices for cardiovascular risk assessment has been comprehensively elucidated in prior research. Large prospective cohort studies spanning distinct ethnic and geographical regions have consistently verified the cardiovascular associations of composite TyG-related indices. Specifically, the large-scale Chinese community-based Kailuan cohort enrolling over 100,000 East Asian adults with a 10-year average follow-up, the multinational European UK Biobank cohort comprising more than 470,000 Caucasian participants followed for a median of 12 years, and the Korean Genome and Epidemiology Study (KoGES) Ansan–Ansung cohort recruiting over 10,000 non-diabetic middle-aged and older residents with 14 years of mean follow-up all demonstrated independent associations of TyG-BMI, TyG-WC and TyG-WHtR with cardiovascular events incidence (31–33).Across these diverse general population cohorts, elevated values of these indices were consistently linked to heightened cardiovascular disease risk. Furthermore, evidence from the study by Park et al. indicated that among non-diabetic individuals, TyG-WC and TyG-WHtR exhibited superior cardiovascular risk predictive capacity compared with isolated TyG and TyG-BMI (33).Subsequent studies extended and refined these findings to vulnerable high-risk populations with CKM syndrome. In the GOLD-Health cohort consisting of older community-dwelling individuals with stage 0–3 CKM syndrome with a median follow-up of 7.2 years, Wang et al. identified significant correlations between the aforementioned composite indices and cardiovascular disease incidence, and TyG-WC manifested the strongest independent association (34). Consistent evidence was also reported by Hong et al. in the China Health and Retirement Longitudinal Study(CHARLS) cohort involving multi-region middle-aged and older Chinese adults followed over a median period of 9 years, revealing that per SD increment, TyG-BMI, TyG-WC, TyG-WHtR conferred greater increments in cardiovascular risk relative to TyG, among which TyG-WC displayed the most prominent effect size (12).

In the present study, TyG-related indices incorporating central adiposity, particularly TyG-WC and TyG-WHtR, demonstrated stronger associations with cardiovascular events compared with TyG and TyG-BMI, highlighting the added value of integrating fat distribution into insulin resistance assessment. Notably, after full multivariable adjustment, TyG was no longer independently associated with cardiovascular outcomes, suggesting that isolated glyco-lipid dysregulation may be insufficient to capture the complexity of cardiometabolic risk without consideration of adiposity patterns. When different exposure patterns were evaluated, both baseline levels and cumulative exposure of TyG-related indices were significantly associated with cardiovascular risk, with cumulative exposure exhibiting more pronounced associations. In contrast, longitudinal mean changes in most indices were not significantly associated with outcomes, except for TyG-BMI, potentially reflecting the limited ability of short-term fluctuations to capture cumulative metabolic insults. Taken together, these findings support the value of baseline and cumulative TyG-related indices—particularly those incorporating central adiposity(TyG-WC and TyG-WHtR)—as practical and scalable tools for cardiovascular risk stratification in individuals with CKM syndrome.

The stronger associations observed for TyG-WC and TyG-WHtR may be explained by their ability to simultaneously capture IR and central adiposity, thereby more comprehensively reflecting the combined burden of metabolic dysfunction and visceral fat accumulation. Central obesity is more strongly associated with cardiovascular disease than generalized obesity, primarily owing to the distinct biological properties of visceral adipose tissue (35).Visceral adipose tissue exhibits substantially greater macrophage infiltration than subcutaneous fat, and secretes elevated levels of pro-inflammatory cytokines including tumor necrosis factor-αand interleukin-6 while suppressing adiponectin secretion, thereby driving endothelial injury and atherosclerotic progression. In addition, free fatty acids released from visceral fat impair hepatic and skeletal muscle insulin signaling, predisposing individuals to systemic insulin resistance. Central obesity further predisposes to ectopic fat deposition within epicardial, myocardial and perivascular compartments, which contributes to cardiomyocyte dysfunction, left ventricular remodeling and deteriorated cardiac systolic function. Via paracrine secretion of bioactive mediators, perivascular adipose tissue directly augments vascular stiffness and exacerbates atherosclerotic plaque instability, consequently mediating target organ damage. Furthermore, visceral adipose tissue constitutes a primary source of plasminogen activator inhibitor-1 (PAI-1), which attenuates fibrinolytic capacity and elevates thrombotic risk, and synergizes with inflammatory mediators to promote cardiovascular disease pathogenesis.

In terms of predictive performance, baseline and cumulative TyG-WC demonstrated the highest C-index, supporting their relatively better discriminative ability for cardiovascular events in individuals with CKM syndrome. TyG-WC also showed a modest but statistically significant advantage over TyG-WHtR. Notably, baseline and cumulative measures exhibited similar predictive performance and both clearly outperformed mean change metrics, underscoring the greater prognostic relevance of sustained metabolic burden over short-term variation. Consistent with our findings, Park et al. demonstrated that TyG-WC and TyG-WHtR yielded significantly higher C-index values than the original TyG index and TyG-BMI (33). Based on data from the National Health and Nutrition Examination Survey (NHANES), Dang K et al. and Zheng D et al. consistently reported that TyG-WC and TyG-WHtR outperformed TyG-BMI and TyG in identifying cardiovascular disease in the general U.S. population (36, 37). Similarly, Xia X et al. reported in the Chinese Kailuan cohort that TyG-related indices incorporating adiposity showed improved discriminatory performance for atherosclerotic cardiovascular disease compared with TyG alone (31). In the GOLD-health cohort, Wang M et al. also observed that TyG-WC exhibited the strongest predictive capacity among insulin resistance-related indices in older adults with CKM syndrome (34).Collectively, these studies consistently indicate that TyG-related indices incorporating adiposity, particularly TyG-WC and TyG-WHtR, provide relatively better discriminatory performance compared with TyG or TyG-BMI; however, the magnitude of improvement in C-statistics is generally modest, suggesting that their incremental predictive value beyond traditional risk factors remains limited. Future studies incorporating additional clinical variables, biomarkers, and advanced modeling approaches, including machine learning techniques, are warranted to further improve predictive performance.

In terms of model fit, compared with the base model, models incorporating cumulative measures of TyG, TyG-BMI, TyG-WC, and TyG-WHtR demonstrated improved model fit, as indicated by lower AIC and BIC values. Among these, cumulative TyG-WC conferred the greatest improvement in model fit, followed by TyG-WHtR. In addition, cumulative exposure models relatively showed better fit than those based on baseline measurements alone, whereas annual mean change metrics provided poor incremental improvement. These findings suggest that baseline and cumulative TyG-WC may aid in the identification of older adults with CKM syndrome who are at elevated risk of incident cardiovascular events, with baseline TyG-WC representing a simpler and more practical tool that showed predictive utility comparable to cumulative TyG-WC. Cho et al. demonstrated that TyG-WC is a superior predictor of cardiovascular disease risk compared with other insulin resistance-related indices, such as the homeostasis model assessment, TyG, and TyG-BMI (38). Taken together, our findings suggest that TyG-WC—particularly when assessed cumulatively, with baseline assessment offering comparable utility—may serve as an auxiliary, pragmatic and scalable tool for refining cardiovascular risk stratification in older adults with CKM syndrome, although it cannot replace conventional cardiovascular risk-assessment instruments. In resource-limited rural health systems, single baseline measurements are clinically feasible for routine risk screening, while repeated assessments for cumulative exposure calculation offer incremental predictive value and should not be overlooked.

Our study confirms that the risk of cardiovascular events increases progressively with advancing CKM syndrome stages, consistent with CKM syndrome as a progressive multisystem disorder linking metabolic dysfunction, kidney injury, and cardiovascular damage. AHA defines CKM syndrome as a continuum ranging from isolated metabolic risk to subclinical cardiovascular disease, highlighting the accumulation of vascular injury with disease progression. In our rural elderly population, the stepwise increase in cardiovascular risk likely reflects worsening IR, chronic low-grade inflammation, endothelial dysfunction, and accelerated atherosclerosis as CKM stages advance. These findings underscore the importance of early identification and intervention, particularly among individuals in early CKM stages, to halt disease progression and reduce cardiovascular risk in underserved rural populations with limited access to specialized care. Currently, although the precise biological mechanisms linking elevated TyG-related indices to increased cardiovascular risk in individuals with CKM syndrome stages 0–3 remain incompletely understood, these indices serve as reliable surrogate markers of IR and may capture the integrated effects of glucose-lipid dysregulation and adiposity-related metabolic burden. The molecular underpinnings of CKM syndrome involve a complex network of interrelated mechanisms, including hyperglycemia, overactivation of the renin-angiotensin-aldosterone system (RAAS), oxidative stress, lipotoxicity, mitochondrial dysfunction, impaired calcium handling, and chronic low-grade inflammation (39). These pathways interact and converge to promote vascular injury and accelerate cardiovascular disease progression. In individuals with CKM syndrome, IR not only reflects underlying metabolic dysfunction but also actively exacerbates these pathological processes. Mechanistically, IR impairs insulin signaling via the Phosphoinositide 3-kinase/AKT Signaling Pathway(PI3K/Akt) pathway, leading to reduced nitric oxide bioavailability, while simultaneously enhancing activation of the Mitogen-Activated Protein Kinases(MAPK) pathway. These alterations promote vasoconstriction and pro-inflammatory responses, increase endothelin-1 activity, and contribute to endothelial dysfunction, which may further drive downstream myocardial remodeling, stiffness, and structural injury (40, 41).These mechanisms may partly explain the stronger associations observed for TyG-related indices incorporating central adiposity—in predicting cardiovascular risk in this population.

Beyond the cardiovascular risk gradient described above, we observed heterogeneity in the incremental predictive value of TyG-related indices differed across CKM syndrome stages. In analyses of overall follow-up, TyG-related indices provided greater predictive performance in CKM syndrome stages 0–1 than in stages 2–3, supporting their potential for early risk identification. Several interconnected mechanisms may underpin this finding. First, this pattern reflects the spectrum effect (42): the high prevalence of hypertension, diabetes, dyslipidemia, and renal dysfunction among those with advanced CKM syndrome (1), which restricts the range of traditional risk predictors, thereby limiting the incremental discrimination of a single metabolic biomarker. The predictive value of biomarkers is therefore population-specific and may not generalize across risk strata. Second, participants in later CKM syndrome stages are more likely to receive intensive antihypertensive, glucose-lowering, and lipid-lowering pharmacotherapy, which may attenuate the underlying association between TyG-related indices and cardiovascular outcomes through treatment-related risk modification. Third, IR dominates cardiovascular risk in early CKM syndrome, whereas in advanced stages, established multi-organ injury and the convergence of heterogeneous pathogenic mechanisms dilute the relative contribution of any single biomarker. This is consistent with the CKM syndrome framework, which conceptualizes a progressive trajectory from metabolic dysregulation to established organ damage. With respect to association strength, most annual mean change indicators were not associated with cardiovascular events; only the annual mean change in TyG-BMI exhibited a weak association. This was also the only indicator demonstrating a statistically significant time-to-CVD interaction in longitudinal trajectory analysis. This differential finding is most likely attributable to the essential differences in metabolic characteristics of the study populations. Specifically, in this cohort of rural older adults, TyG-WC and TyG-WHtR exhibit relatively limited year-to-year variability or a narrower range of fluctuation over the follow-up period. Consequently, the magnitude of longitudinal change in TyG-WC and TyG-WHtR may have been too subtle to yield sufficient statistical power or to translate into meaningful divergence in cardiovascular risk trajectories. Nevertheless, all annual mean change indicators overall yielded inferior predictive performance relative to baseline and cumulative indicators.

### 4.1 Study strengths and limitations

This study possesses several notable strengths. First, based on longitudinal health examination data derived from rural elderly Chinese individuals, this study adopted an integrated analytical framework encompassing static baseline measurements, cumulative exposure indices, and longitudinal annual mean change analysis. To our knowledge, this represents a comprehensive and systematic study exploring the associations of TyG-related obesity indices with incident cardiovascular events among adults aged ≥65 years with CKM syndrome stages 0–3. Second, the utilization of data from the National Basic Public Health Service Project, which provides annual standardized health assessments for rural older adults, demonstrates the feasibility of leveraging existing public health infrastructure for cardiovascular risk screening. Third, the inclusion of multiple TyG-related indices (TyG-BMI, TyG-WC, and TyG-WHtR) and the systematic comparison of baseline, cumulative, and annual mean change metrics provide valuable insights into the optimal application of these indices in clinical practice. Fourth, the rigorous statistical methodology, including AFT models, RCS analyses, TD-AUC, and comprehensive sensitivity analyses using both PMM and CART multiple imputation, enhances the robustness and reliability of our findings. These findings furnish a clinically applicable and population-tailored strategy for the implementation of TyG-related obesity composite indices in cardiovascular disease prevention targeted at the early-stage CKM population in rural China.

Several limitations should be acknowledged. First, although we analyzed baseline values, cumulative exposure, and annual mean changes under the available data structure, future studies with more frequent repeated measurements would enable more detailed characterization of dynamic trajectories and their prognostic implications. The 3-year exposure assessment period, while adequate for capturing intermediate-term changes, may have been insufficient to detect subtle longitudinal variations in TyG-WC and TyG-WHtR. Second, cardiovascular events were ascertained from self-reported physician diagnoses without formal medical chart review, which introduces risks of recall bias and outcome misclassification. A validation study by Sabanayagam et al. reported a low false-positive rate for self-reported CVD, implying limited impact of false-positive misclassification (43). Even so, some true events may go undetected. If misclassification is non-differential by exposure status, effect estimates tend to be attenuated toward the null (44); accordingly, observed positive associations are unlikely to be inflated and may be conservative. Exposure assessment was independent of outcome ascertainment, and follow-up protocols were consistent across exposure groups, making substantial differential misclassification unlikely. Nonetheless, owing to imperfect sensitivity of this ascertainment approach, absolute event rates should be interpreted as minimum estimates. Future studies with centralized medical-record adjudication or multi-source outcome verification are warranted to validate and extend these findings. Third, the observational design limits causal inference; randomized trials are needed to determine whether interventions targeting the TyG-related index directly reduce cardiovascular events. Fourth, despite adjustment for major confounders, residual confounding from unmeasured variables including medication use, physical activity, and dietary patterns cannot be fully excluded. Omission of these variables may bias effect estimates away from the null, potentially overestimating the strength of the association. However, a recent prospective cohort study of 282,920 UK Biobank participants with CKM syndrome stages 0–3 reported that TyG-related indices remained significantly associated with cardiovascular disease after joint adjustment for physical activity and diet quality (32), indicating that lifestyle-related residual confounding, though present, cannot fully account for the observed associations. Fifth, our findings were obtained from a single region cohort in rural Huai’an, China, and the study sample was limited to rural elderly residents. Accordingly, these results may not be directly applicable to other populations. Further validation is needed to assess whether these findings can be generalized to other populations. Populations in different regions may have different risk factors and prevalence of disease because of differences in genetics, environment, lifestyle, and health care systems. Further validation in other geographic and ethnic settings is warranted to assess generalizability.

### 4.2 Clinical implications

Our findings carry important implications for cardiovascular prevention among rural older adults with early CKM syndrome stages. First, with respect to disease staging, the graded increment in cardiovascular risk across successive CKM stages underscores the value for targeted risk identification. This stepwise progression highlights the importance of early screening and intervention to prevent progression to advanced stages and reduce cardiovascular morbidity and mortality. Second, regarding risk stratification tools, baseline and cumulative TyG-WC serves as a simple, low-cost biomarker well-adapted to resource-constrained rural primary care settings. Its calculation requires only routine laboratory measurements (FPG and TG) and WC, with no need for specialized or sophisticated equipment. This characteristic makes baseline and cumulative TyG-WC particularly suitable for implementation in underserved rural areas where access to advanced diagnostic technologies is limited. Third, baseline TyG-WC demonstrated equivalent predictive performance for incident cardiovascular events to cumulative TyG-WC, indicating that a single measurement yields equivalent utility for 6-year risk stratification in this cohort of rural older adults with relatively stable metabolic phenotypes. This equivalence alleviates the burden of repeated follow-up in resource-limited settings and enables timely risk stratification at the initial clinical encounter. Nevertheless, while a single baseline measurement may suffice for general risk prediction, cumulative exposure of TyG-derived indices still provides critical complementary prognostic information. Long-term monitoring of these indices remains vital for cardiovascular disease prevention, particularly in populations with dynamic metabolic profiles. Fourth, the non-linear associations identified for baseline TyG-WC suggest that cardiovascular risk may increase more steeply at higher TyG-WC levels, with an estimated inflection point of 728.66 in this cohort. However, this value should be interpreted with considerable caution, as it was a model-derived estimate from a single cohort, subject to statistical uncertainty (e.g., knot placement and sample size), and does not represent a clinically validated threshold. Accordingly, it should not be extrapolated or used as a clinical cut-off to guide individual risk assessment or treatment decisions. External validation in independent cohorts, preferably with diverse demographic and clinical profiles, is essential to determine whether this inflection point remains stable and clinically meaningful before any translational application is considered. Fifth, the relatively better discriminative performance of baseline and cumulative TyG-WC compared with TyG and TyG-BMI suggests that these measures may be preferable auxiliary tools among the TyG-related indices. When selecting among these indices for cardiovascular risk stratification, baseline and cumulative TyG-WC could be considered as first-line TyG-related options in settings where central obesity is highly prevalent, although their overall discriminative ability remains moderate, and these indices should not replace conventional risk-assessment instruments.

## 5 Conclusion

In conclusion, this study provides comprehensive evidence that baseline and cumulative TyG-related indices, particularly those incorporating central adiposity (TyG-WC and TyG-WHtR), are independent risk factors and demonstrate moderate predictive value for cardiovascular events in rural older adults with CKM syndrome stages 0–3 in China. The robust associations and modest discriminative performance of baseline and cumulative TyG-WC underscore their potential as simple, low-cost, and clinically feasible auxiliary tools for cardiovascular risk stratification in resource-limited primary care settings, although they cannot be regarded as a replacement for conventional cardiovascular risk-assessment instruments. Notably, these indices can be readily obtained from longitudinal health examination data collected through the National Basic Public Health Service Project, which provides annual standardized assessments for rural older adults aged ≥65 years. These findings suggest that TyG-related obesity composite indices may be considered as adjunctive tools in cardiovascular risk assessment, particularly for early-stage CKM populations in rural China, and highlight the value of leveraging existing public health infrastructure for cardiovascular disease prevention; however, further validation is needed before their routine integration can be recommended.

## Data Availability

The datasets presented in this article are not readily available because this retrospective cohort study uses clinical data collected from four primary healthcare centers in Huai’an City, Jiangsu Province, China. Raw clinical data containing sensitive patient information are not publicly available due to privacy regulations and institutional ethical review requirements. De-identified datasets can be accessed upon reasonable request from the corresponding author, subject to approval by the relevant healthcare institutions and ethics committee. Requests to access the datasets should be directed to yuanxiaodan107@163.com.

## Glossary

AHA: American Heart Association
CKM: Cardiovascular-kidney-metabolic
CVD: Cardiovascular disease
IR: Insulin resistance
TyG: Triglyceride-glucose index
TyG-BMI: Triglyceride-glucose index-body mass index
TyG-WC: Triglyceride-glucose index-waist circumference index
TyG-WHtR: Triglyceride-glucose index-waist-to-height ratio index
FPG: Fasting plasma glucose
TG: Triglycerides
HDL-C: High-density lipoprotein cholesterol
LDL-C: Low-density lipoprotein cholesterol
BMI: Body mass index
WC: Waist circumference
WHtR: waist-to-height ratio
SBP: Systolic blood pressure
DBP: Diastolic blood pressure
TC: Total cholesterol
Cr: Creatinine
eGFR: Estimated glomerular filtration rate
CKD: Chronic Kidney Disease
CKD-EPI: Chronic Kidney Disease Epidemiology Collaboration
MICE: Multiple imputation by chained equations
PMM: Predictive mean matching
CART: Classification and regression trees
AFT: Accelerated failure time
PH: proportional hazards
TRs: Time ratios
TR: Time ratio
AIC: Akaike information criterion
SD: Standard deviation
IQR: Interquartile range
VIF: Variance inflation factor
RCS: Restricted cubic spline
LMM: Linear mixed-effects models
SE: Standard errors
TD-ROC: Time-dependent receiver operating characteristic
AUC: Area under the curve
TD-AUC: Time-dependent AUC
BH: Benjamini-Hochberg
FDR: False discovery rate
BIC: Bayesian information criterion
LRT: Likelihood ratio test
95%CI 95%: confidence intervals
KoGES: Korean Genome and Epidemiology Study
GOLD-Health: Guangzhou Older Longitudinal Dynamic Health cohort
CHARLS: China Health and Retirement Longitudinal Study
NHANES: National Health and Nutrition Examination Survey
PAI-1: Plasminogen activator inhibitor-1
RAAS: Renin-angiotensin-aldosterone system
PI3K/Akt: Phosphoinositide 3-kinase/Akt signaling pathway
MAPK: Mitogen-Activated Protein Kinases pathway
