# Supplementary material for: Predictive value of baseline, cumulative exposure and annual mean changes in TyG-related indices for cardiovascular events in older rural adults with cardiovascular-kidney-metabolic syndrome stages 0–3: a cohort study
Source: Front Endocrinol (Lausanne). 2026 Sep 30;17:1952253. doi: 10.3389/fendo.2026.1952253 (PMC13624300; doi:10.3389/fendo.2026.1952253)
Supplement: Supplementary file 1 [file SupplementaryFile1.docx]

**Supplementary Materials**

**Predictive Value of Baseline, Cumulative Exposure and Annual Mean Changes in TyG-related Indices for Cardiovascular Events in Older Rural Adults with Cardiovascular-Kidney-Metabolic Syndrome Stages 0–3: A Cohort Study**

Yangzhen Xue^1†^, Qingxiu Tian^2†^, Rong Zhang ^1^ ,Jiayou Chu^1^, Guilin Liu^1^, Huishu Sun^1^, Yong Qin^3*^, Xiaodan Yuan^4*^

^1^School of Nursing, Nanjing University of Chinese Medicine, Nanjing, Jiangsu Province, China

^2^Department of Nutrition, Xuyi County T.C.M. Hospital, Huai’an, Jiangsu Province, China

^3^Department of Anesthesiology, Xuyi County T.C.M. Hospital, Huai’an, Jiangsu Province, China

^4^Department of Public Health, Affiliated Hospital of Integrated Traditional Chinese and Western Medicine, Nanjing University of Chinese Medicine, Nanjing, Jiangsu Province, China

† These authors contributed to the work equally and should be regarded as the co-first authors.

* These authors contributed to the work equally and should be regarded as the co-corresponding authors.

**Correspondence:**

Yong Qin, Email: 2214386865@qq.com;

Xiaodan Yuan, Email: yuanxiaodan107@163.com

[Table S1. Definition of cardiovascular-kidney-metabolic (CKM) syndrome stages	4](#_Toc16570)

[TableS2. The Akaike information criterion (AIC) of different distributions for AFT models(data imputed with PMM)	5](#_Toc5177)

[TableS3. The Akaike information criterion (AIC) of different distributions for AFT models(data imputed with CART)	6](#_Toc4371)

[TableS4. The Akaike information criterion (AIC) of different distributions for AFT models(original non-imputed data)	7](#_Toc5876)

[TableS5. Time ratio of associations between baseline, cumulative and annual mean change TyG-related indices with cardiovascular events among elderly rural residents with CKM syndrome stages 0–3（data imputed with CART）	15](#_Toc10420)

[TableS6. Time ratio of associations between baseline, cumulative and annual mean change TyG-related indices with cardiovascular events among elderly rural residents with CKM syndrome stages 0–3（original non-imputed data）	20](#_Toc32567)

[TableS7. Predictive value of cardiovascular events prediction model(original non-imputed data)	24](#_Toc13976)

[TableS8. Comparison and incremental predictive value of cardiovascular events prediction model(data imputed with CART)	25](#_Toc1317)

[TableS9. Comparison and incremental predictive value of cardiovascular events prediction model(original non-imputed data)	26](#_Toc2847)

[TableS10. Pairwise comparison of cardiovascular disease prediction models based on baseline TyG-related indices(data imputed with PMM)	27](#_Toc30019)

[TableS11. Pairwise comparison of cardiovascular disease prediction models based on 2-year cumulative TyG-related indices(data imputed with PMM)	28](#_Toc4050)

[TableS12. Pairwise comparison of cardiovascular disease prediction models based on 3-year cumulative TyG-related indices(data imputed with PMM)	29](#_Toc3490)

[TableS13. Pairwise comparison of cardiovascular disease prediction models based on 3-year mean change TyG-related indices(data imputed with PMM)	30](#_Toc25194)

[TableS14. Pairwise comparison of cardiovascular disease prediction models based on TyG indices(data imputed with PMM)	31](#_Toc5470)

[TableS15. Pairwise comparison of cardiovascular disease prediction models based on TyG-BMI indices(data imputed with PMM)	32](#_Toc21706)

[TableS16. Pairwise comparison of cardiovascular disease prediction models based on TyG-WC Indices(data imputed with PMM)	33](#_Toc2332)

[TableS17. Pairwise comparison of cardiovascular disease prediction models based on TyG-WHtR Indices(data imputed with PMM)	34](#_Toc25103)

[TableS18. Pairwise comparison of cardiovascular disease prediction models based on baseline TyG-related indices(data imputed with CART)	35](#_Toc19564)

[TableS19. Pairwise comparison of cardiovascular disease prediction models based on 2-year cumulative TyG-related indices(data imputed with CART)	36](#_Toc26855)

[TableS20. Pairwise comparison of cardiovascular disease prediction models based on 3-year cumulative TyG-related indices(data imputed with CART)	37](#_Toc3891)

[TableS21. Pairwise comparison of cardiovascular disease prediction models based on 3-year mean change TyG-related indices(data imputed with CART)	38](#_Toc15814)

[TableS22. Pairwise comparison of cardiovascular disease prediction models based on TyG indices(data imputed with CART)	39](#_Toc20373)

[TableS23. Pairwise comparison of cardiovascular disease prediction models based on TyG-BMI indices(data imputed with CART)	40](#_Toc31678)

[TableS24. Pairwise comparison of cardiovascular disease prediction models based on TyG-WC indices(data imputed with CART)	41](#_Toc25586)

[TableS25. Pairwise comparison of cardiovascular disease prediction models based on TyG-WHtR indices(data imputed with CART)	42](#_Toc28922)

[TableS26. Comparison results of AUCs across time points for different models (data imputed with PMM)	43](#_Toc5612)

[TableS27. Comparison results of AUCs across time points for different models (data imputed with CART)	45](#_Toc11307)

[Fig.S1 Kaplan-Meier curves for cardiovascular events cumulative incidence by CKM syndrome stages 0–3(data imputed with CART).	46](#_Toc24217)

[Fig.S2 Kaplan-Meier curves for cardiovascular events cumulative incidence by CKM syndrome stages 0–3(original non-imputed data).	47](#_Toc6777)

[Fig.S3 RCS analyses between baseline TyG-related indices and cardiovascular events in populations with CKM syndrome stages 0–3(data imputed with CART).	48](#_Toc5454)

[Fig.S4 RCS analyses between baseline TyG-related indices and cardiovascular events in populations with CKM syndrome stages 0–3(original non-imputed data).	49](#_Toc24993)

[Fig.S5 Predictive performance of TyG-related indices for 4-year and 5-year cardiovascular events in populations with CKM syndrome stages 0–3(data imputed with PMM).	50](#_Toc11680)

[Fig.S6 Predictive performance of TyG-related indices for cardiovascular events in populations with CKM syndrome stages 0–3(data imputed with CART)	51](#_Toc16495)

[Fig.S7 Predictive performance of TyG-related indices for 4-year and 5-year cardiovascular events in populations with CKM syndrome stages 0–3(data imputed with CART).	52](#_Toc20414)

[Fig.S8 Longitudinal trajectories of TyG-related indices in populations with CKM syndrome stages 0–3 stratified by CVD status(data imputed with CART).	53](#_Toc7160)

**Table S1. Definition of cardiovascular-kidney-metabolic (CKM) syndrome stages**

| CKM syndrome stages | Definition |
| --- | --- |
| Stage 0: No CKM risk factors | Individuals with normal BMI and WC, normoglycemia, normotension, a normal lipid profile, and no evidence of CKD or subclinical/clinical CVD. |
| Stage 1: Excess of dysfunctional adiposity | Individuals with overweight/obesity, abdominal obesity, or dysfunctional adipose tissue, without the presence of other metabolic risk factors or CKD.  BMI ≥ 23 kg/m^2^, WC≥80/90 cm in women/ men, or FPG level 100-125 mg/dL. |
| Stage 2: Metabolic diseases or moderate-to-high-risk CKD | Presence of hypertension , diabetes , metabolic syndrome , high triglyceride levels , or moderate-high risk CKD(30≤eGFR<60 mL/min/1.73 m^2^). |
| Stage 3: Subclinical CVD in CKM syndrome | With a Predicting Risk of Cardiovascular Disease Events (PREVENT) score indicating a 10-year total CVD risk ≥20% or very high-risk CKD (eGFR<30 mL/min/1.73 m^2^) . |
| Stage 4: Clinical CVD in in CKM syndrome | Occurrence of coronary heart disease, heart failure, stroke, peripheral artery disease, or atrial fibrillation, and is further subdivided into stage 4a (without kidney failure) and stage 4b (with kidney failure). |
| In this study, the definition of CKM syndrome was adapted from the criteria proposed in the 2023 AHA Presidential Advisory on CKM Health[1], with several key considerations tailored to the characteristics of our study cohort.  • For the glycemic definition of CKM syndrome stage 1, only the FPG criterion (100–125 mg/dL) consistent with the AHA was applied in this study, given the absence of HbA1c measurements in our cohort.  • For CKM syndrome stages 2–3, CKD was defined by the CKD-EPI equation in line with the AHA criteria[1]. As ACR was not measured in this cohort, we used only CKD-EPI–based eGFR for CKM staging, rather than the KDIGO classification (which requires ACR).  • We lacked diagnostic examinations for subclinical atherosclerosis or heart failure (coronary angiography, coronary CT angiography, cardiac biomarkers, and echocardiographic parameters). Therefore, we used the PREVENT risk prediction model as a risk equivalent for defining subclinical CVD[2].  Abbreviations: CKM, cardiovascular-kidney-metabolic syndrome; CKD, chronic kidney disease; CVD, cardiovascular disease; BMI, body mass index; WC, waist circumference; FPG, fasting plasma glucose; PREVENT, Predicting Risk of Cardiovascular Disease Events; eGFR, estimated glomerular filtration rate; AHA, American Heart Association; HbA1c, hemoglobin A1c; CKD-EPI, Chronic Kidney Disease Epidemiology Collaboration; KDIGO, Kidney Disease Improving Global Outcomes; ACR, albumin-creatinine ratio. | |

**TableS2.** The Akaike information criterion (AIC) of different distributions for AFT models(data imputed with PMM)

| Indicators | Model | AIC | |
| --- | --- | --- | --- |
|  |  | weibull | lognormal |
| Baseline TyG | Model 1 | 10269.78 | 10257.95 |
|  | Model 2 | 10255.55 | 10242.22 |
|  | Model 3 | 10226.92 | 10212.45 |
| Baseline TyG-BMI | Model 1 | 10265.04 | 10254.45 |
|  | Model 2 | 10251.47 | 10239.40 |
|  | Model 3 | 10223.48 | 10210.54 |
| Baseline TyG-WC | Model 1 | 10247.57 | 10237.48 |
|  | Model 2 | 10233.33 | 10221.16 |
|  | Model 3 | 10207.47 | 10195.00 |
| Baseline TyG-WHtR | Model 1 | 10250.17 | 10239.17 |
|  | Model 2 | 10240.21 | 10228.05 |
|  | Model 3 | 10213.61 | 10200.93 |
| 2-year cumulative TyG | Model 1 | 10266.03 | 10255.25 |
|  | Model 2 | 10251.81 | 10239.80 |
|  | Model 3 | 10224.29 | 10210.88 |
| 2-year cumulative TyG-BMI | Model 1 | 10259.90 | 10250.20 |
|  | Model 2 | 10246.30 | 10235.22 |
|  | Model 3 | 10220.18 | 10207.92 |
| 2-year cumulative TyG-WC | Model 1 | 10238.05 | 10229.93 |
|  | Model 2 | 10223.29 | 10213.07 |
|  | Model 3 | 10200.14 | 10189.59 |
| 2-year cumulative TyG-WHtR | Model 1 | 10242.32 | 10232.75 |
|  | Model 2 | 10232.93 | 10222.42 |
|  | Model 3 | 10208.56 | 10197.33 |
| 3-year cumulative TyG | Model 1 | 10264.48 | 10253.93 |
|  | Model 2 | 10250.32 | 10238.63 |
|  | Model 3 | 10223.75 | 10210.52 |
| 3-year cumulative TyG-BMI | Model 1 | 10256.35 | 10246.64 |
|  | Model 2 | 10242.41 | 10231.52 |
|  | Model 3 | 10217.73 | 10205.75 |
| 3-year cumulative TyG-WC | Model 1 | 10234.82 | 10227.11 |
|  | Model 2 | 10219.24 | 10209.99 |
|  | Model 3 | 10197.21 | 10187.26 |
| 3-year cumulative TyG-WHtR | Model 1 | 10239.86 | 10230.54 |
|  | Model 2 | 10230.16 | 10220.35 |
|  | Model 3 | 10206.58 | 10195.79 |
| 3-year mean change TyG | Model 1 | 10270.24 | 10257.59 |
|  | Model 2 | 10256.18 | 10241.91 |
|  | Model 3 | 10227.27 | 10212.51 |
| 3-year mean change TyG-BMI | Model 1 | 10266.21 | 10252.65 |
|  | Model 2 | 10251.14 | 10235.55 |
|  | Model 3 | 10222.54 | 10207.22 |
| 3-year mean change TyG-WC | Model 1 | 10270.53 | 10258.08 |
|  | Model 2 | 10256.37 | 10242.34 |
|  | Model 3 | 10227.11 | 10212.47 |
| 3-year mean change TyG-WHtR | Model 1 | 10270.53 | 10258.07 |
|  | Model 2 | 10256.40 | 10242.33 |
|  | Model 3 | 10227.16 | 10212.46 |

AFT, accelerated failure time; PMM, predictive mean matching; other abbreviations as in Table 1. Model I was unadjusted; Model II was adjusted for age, gender, smoking status and drinking status; Model III was adjusted for age, gender, smoking status, drinking status, SBP, diabetes, hypertension and LDL-C.

**TableS3.** The Akaike information criterion (AIC) of different distributions for AFT models(data imputed with CART)

| **Indicators** | **Model** | **AIC** | |
| --- | --- | --- | --- |
|  |  | **weibull** | **lognormal** |
| Baseline TyG | Model 1 | 10269.69 | 10257.79 |
|  | Model 2 | 10255.42 | 10242.06 |
|  | Model 3 | 10227.52 | 10213.20 |
| Baseline TyG-BMI | Model 1 | 10263.96 | 10253.64 |
|  | Model 2 | 10250.38 | 10238.60 |
|  | Model 3 | 10223.27 | 10210.31 |
| Baseline TyG-WC | Model 1 | 10248.20 | 10238.41 |
|  | Model 2 | 10234.18 | 10221.93 |
|  | Model 3 | 10208.61 | 10195.79 |
| Baseline TyG-WHtR | Model 1 | 10250.92 | 10239.93 |
|  | Model 2 | 10240.84 | 10228.60 |
|  | Model 3 | 10214.73 | 10201.79 |
| 2-year cumulative TyG | Model 1 | 10265.88 | 10254.93 |
|  | Model 2 | 10251.68 | 10239.45 |
|  | Model 3 | 10225.16 | 10211.65 |
| 2-year cumulative TyG-BMI | Model 1 | 10259.08 | 10249.39 |
|  | Model 2 | 10245.63 | 10234.38 |
|  | Model 3 | 10219.49 | 10207.25 |
| 2-year cumulative TyG-WC | Model 1 | 10240.20 | 10232.25 |
|  | Model 2 | 10225.18 | 10214.93 |
|  | Model 3 | 10202.02 | 10191.33 |
| 2-year cumulative TyG-WHtR | Model 1 | 10244.27 | 10234.80 |
|  | Model 2 | 10234.64 | 10223.92 |
|  | Model 3 | 10210.56 | 10199.18 |
| 3-year cumulative TyG | Model 1 | 10264.42 | 10253.88 |
|  | Model 2 | 10250.18 | 10238.62 |
|  | Model 3 | 10223.99 | 10210.93 |
| 3-year cumulative TyG-BMI | Model 1 | 10255.81 | 10246.26 |
|  | Model 2 | 10241.98 | 10231.00 |
|  | Model 3 | 10216.78 | 10204.97 |
| 3-year cumulative TyG-WC | Model 1 | 10237.10 | 10229.83 |
|  | Model 2 | 10221.55 | 10211.97 |
|  | Model 3 | 10198.83 | 10189.17 |
| 3-year cumulative TyG-WHtR | Model 1 | 10241.75 | 10232.71 |
|  | Model 2 | 10231.97 | 10221.92 |
|  | Model 3 | 10208.33 | 10197.80 |
| 3-year mean change TyG | Model 1 | 10269.80 | 10256.94 |
|  | Model 2 | 10255.88 | 10241.46 |
|  | Model 3 | 10227.44 | 10212.54 |
| 3-year mean change TyG-BMI | Model 1 | 10265.38 | 10251.75 |
|  | Model 2 | 10250.17 | 10234.52 |
|  | Model 3 | 10222.49 | 10206.20 |
| 3-year mean change TyG-WC | Model 1 | 10269.93 | 10257.23 |
|  | Model 2 | 10255.28 | 10240.95 |
|  | Model 3 | 10226.18 | 10211.16 |
| 3-year mean change TyG-WHtR | Model 1 | 10270.02 | 10257.28 |
|  | Model 2 | 10255.37 | 10241.03 |
|  | Model 3 | 10226.22 | 10211.16 |

AFT, accelerated failure time; CART,classification and regression trees; other abbreviations as in Table 1. Model I was unadjusted; Model II was adjusted for age, gender, smoking status and drinking status; Model III was adjusted for age, gender, smoking status, drinking status, SBP, diabetes, hypertension and LDL-C.

**TableS4.** The Akaike information criterion (AIC) of different distributions for AFT models(original non-imputed data)

| **Indicators** | **Model** | **AIC** | |
| --- | --- | --- | --- |
|  |  | **weibull** | **lognormal** |
| Baseline TyG | Model 1 | 9599.59 | 9588.73 |
|  | Model 2 | 9588.64 | 9575.87 |
|  | Model 3 | 9430.55 | 9417.43 |
| Baseline TyG-BMI | Model 1 | 9598.90 | 9588.46 |
|  | Model 2 | 9588.41 | 9575.85 |
|  | Model 3 | 9430.74 | 9417.38 |
| Baseline TyG-WC | Model 1 | 9582.37 | 9573.37 |
|  | Model 2 | 9572.44 | 9561.23 |
|  | Model 3 | 9416.61 | 9405.06 |
| Baseline TyG-WHtR | Model 1 | 9582.77 | 9573.24 |
|  | Model 2 | 9575.93 | 9564.80 |
|  | Model 3 | 9419.76 | 9408.15 |
| 2-year cumulative TyG | Model 1 | 8664.11 | 8648.65 |
|  | Model 2 | 8654.01 | 8638.34 |
|  | Model 3 | 8512.67 | 8497.45 |
| 2-year cumulative TyG-BMI | Model 1 | 8662.80 | 8647.81 |
|  | Model 2 | 8653.13 | 8637.77 |
|  | Model 3 | 8512.72 | 8497.47 |
| 2-year cumulative TyG-WC | Model 1 | 8630.07 | 8618.39 |
|  | Model 2 | 8619.79 | 8607.14 |
|  | Model 3 | 8482.52 | 8470.38 |
| 2-year cumulative TyG-WHtR | Model 1 | 8632.87 | 8619.92 |
|  | Model 2 | 8625.71 | 8612.77 |
|  | Model 3 | 8487.40 | 8474.77 |
| 3-year cumulative TyG | Model 1 | 5663.17 | 5596.90 |
|  | Model 2 | 5643.42 | 5582.49 |
|  | Model 3 | 5555.39 | 5500.30 |
| 3-year cumulative TyG-BMI | Model 1 | 5653.69 | 5589.66 |
|  | Model 2 | 5633.60 | 5575.47 |
|  | Model 3 | 5549.52 | 5496.13 |
| 3-year cumulative TyG-WC | Model 1 | 5648.03 | 5585.06 |
|  | Model 2 | 5629.05 | 5570.89 |
|  | Model 3 | 5544.86 | 5491.75 |
| 3-year cumulative TyG-WHtR | Model 1 | 5644.68 | 5581.21 |
|  | Model 2 | 5628.39 | 5570.36 |
|  | Model 3 | 5543.86 | 5491.03 |
| 3-year mean change TyG | Model 1 | 6071.37 | 6000.51 |
|  | Model 2 | 6051.72 | 5985.41 |
|  | Model 3 | 5947.44 | 5887.17 |
| 3-year mean change TyG-BMI | Model 1 | 6072.64 | 6001.67 |
|  | Model 2 | 6052.34 | 5985.72 |
|  | Model 3 | 5948.51 | 5888.64 |
| 3-year mean change TyG-WC | Model 1 | 6070.51 | 5999.36 |
|  | Model 2 | 6050.95 | 5984.23 |
|  | Model 3 | 5946.54 | 5886.39 |
| 3-year mean change TyG-WHtR | Model 1 | 6070.42 | 5999.29 |
|  | Model 2 | 6050.87 | 5984.14 |
|  | Model 3 | 5946.47 | 5886.31 |

AFT, accelerated failure time; other abbreviations as in Table 1. Model I was unadjusted; Model II was adjusted for age, gender, smoking status and drinking status; Model III was adjusted for age, gender, smoking status, drinking status, SBP, diabetes, hypertension and LDL-C.

**TableS5.** Time ratio of associations between baseline, cumulative and annual mean change TyG-related indices with cardiovascular events among elderly rural residents with CKM syndrome stages 0–3（data imputed with CART）

| **Exposures** | **Model 1** | | **Model 2** | | **Model 3** | |
| --- | --- | --- | --- | --- | --- | --- |
|  | **TR(95%CI)** | ***P* value** | **TR(95%CI)** | ***P* value** | **TR(95%CI)** | ***P* value** |
| **Baseline TyG** | | | | | | |
| Per SD | 0.993(0.967,1.019) | 0.579 | 0.993(0.968,1.019) | 0.612 | 0.995(0.968,1.022) | 0.709 |
| Q1 | Reference | | Reference | | Reference | |
| Q2 | 1.004(0.930,1.085) | 0.914 | 0.998(0.924,1.077) | 0.957 | 0.997(0.924,1.076) | 0.942 |
| Q3 | 1.008(0.937,1.085) | 0.822 | 1.008(0.937,1.084) | 0.829 | 1.002(0.930,1.079) | 0.965 |
| Q4 | 0.967(0.900,1.040) | 0.365 | 0.968(0.901,1.040) | 0.369 | 0.970(0.900,1.046) | 0.427 |
| *P* for trend | 0.406 | | 0.433 | | 0.493 | |
| **Baseline TyG-BMI** | | | | | | |
| Per SD | 0.973(0.948,0.998) | 0.033 | 0.975(0.950,1.000) | 0.050 | 0.979(0.954,1.006) | 0.126 |
| Q1 | Reference | | Reference | | Reference | |
| Q2 | 1.056(0.981,1.137) | 0.146 | 1.052(0.978,1.132) | 0.173 | 1.054(0.980,1.135) | 0.157 |
| Q3 | 1.009(0.934,1.090) | 0.824 | 1.008(0.933,1.089) | 0.845 | 1.017(0.941,1.099) | 0.670 |
| Q4 | 0.971(0.904,1.043) | 0.415 | 0.977(0.909,1.049) | 0.517 | 0.989(0.918,1.066) | 0.778 |
| *P* for trend | 0.225 | | 0.298 | | 0.583 | |
| **Baseline TyG-WC** | | | | | | |
| Per SD | 0.945(0.922,0.969) | ＜0.001 | 0.944(0.921,0.968) | ＜0.001 | 0.946(0.921,0.971) | ＜0.001 |
| Q1 | Reference | | Reference | | Reference | |
| Q2 | 1.053(0.969,1.145) | 0.221 | 1.042(0.959,1.132) | 0.327 | 1.045(0.962,1.136) | 0.296 |
| Q3 | 0.997(0.925,1.074) | 0.932 | 0.991(0.920,1.067) | 0.802 | 0.990(0.919,1.066) | 0.794 |
| Q4 | 0.885(0.824,0.951) | 0.001 | 0.882(0.821,0.948) | 0.001 | 0.888(0.824,0.956) | 0.002 |
| *P* for trend | ＜0.001 | | ＜0.001 | | ＜0.001 | |
| **Baseline TyG-WHtR** | | | | | | |
| Per SD | 0.947(0.924,0.971) | ＜0.001 | 0.952(0.928,0.977) | ＜0.001 | 0.954(0.928,0.980) | 0.001 |
| Q1 | Reference | | Reference | | Reference | |
| Q2 | 1.006(0.928,1.091) | 0.877 | 1.005(0.928,1.089) | 0.901 | 1.006(0.929,1.090) | 0.878 |
| Q3 | 0.995(0.923,1.071) | 0.885 | 0.996(0.924,1.073) | 0.915 | 0.996(0.924,1.074) | 0.921 |
| Q4 | 0.862(0.801,0.928) | ＜0.001 | 0.873(0.809,0.942) | ＜0.001 | 0.877(0.812,0.948) | 0.001 |
| *P* for trend | ＜0.001 | | ＜0.001 | | 0.001 | |
| **2-year cumulative TyG** | | | | | | |
| Per SD | 0.977(0.953,1.002) | 0.077 | 0.978(0.954,1.004) | 0.095 | 0.983(0.957,1.009) | 0.201 |
| Q1 | Reference | | Reference | | Reference | |
| Q2 | 1.006(0.935,1.083) | 0.868 | 1.007(0.936,1.083) | 0.850 | 1.008(0.937,1.084) | 0.833 |
| Q3 | 0.990(0.919,1.067) | 0.797 | 0.990(0.920,1.067) | 0.800 | 0.989(0.917,1.066) | 0.766 |
| Q4 | 0.917(0.853,0.984) | 0.017 | 0.921(0.857,0.989) | 0.024 | 0.926(0.860,0.997) | 0.042 |
| *P* for trend | 0.012 | | 0.019 | | 0.039 | |
| **2-year cumulative TyG-BMI** | | | | | | |
| Per SD | 0.962(0.938,0.987) | 0.003 | 0.963(0.939,0.988) | 0.004 | 0.970(0.945,0.996) | 0.023 |
| Q1 | Reference | | Reference | | Reference | |
| Q2 | 0.973(0.901,1.049) | 0.472 | 0.969(0.898,1.045) | 0.417 | 0.976(0.906,1.052) | 0.531 |
| Q3 | 0.963(0.893,1.039) | 0.327 | 0.961(0.891,1.036) | 0.302 | 0.973(0.902,1.050) | 0.485 |
| Q4 | 0.906(0.843,0.973) | 0.007 | 0.908(0.845,0.977) | 0.010 | 0.924(0.858,0.995) | 0.037 |
| *P* for trend | 0.007 | | 0.011 | | 0.040 | |
| **2-year cumulative TyG-WC** | | | | | | |
| Per SD | 0.937(0.914,0.961) | ＜0.001 | 0.936(0.912,0.959) | ＜0.001 | 0.940(0.915,0.965) | ＜0.001 |
| Q1 | Reference | | Reference | | Reference | |
| Q2 | 0.998(0.925,1.076) | 0.956 | 0.988(0.917,1.065) | 0.757 | 0.992(0.920,1.068) | 0.825 |
| Q3 | 0.934(0.868,1.005) | 0.067 | 0.930(0.865,1.000) | 0.051 | 0.934(0.868,1.005) | 0.067 |
| Q4 | 0.858(0.798,0.922) | ＜0.001 | 0.854(0.795,0.917) | ＜0.001 | 0.864(0.803,0.930) | ＜0.001 |
| *P* for trend | ＜0.001 | | ＜0.001 | | ＜0.001 | |
| **2-year cumulative TyG-WHtR** | | | | | | |
| Per SD | 0.940(0.916,0.964) | ＜0.001 | 0.944(0.920,0.969) | ＜0.001 | 0.949(0.923,0.975) | ＜0.001 |
| Q1 | Reference | | Reference | | Reference | |
| Q2 | 1.009(0.935,1.088) | 0.821 | 1.012(0.938,1.091) | 0.758 | 1.012(0.939,1.091) | 0.752 |
| Q3 | 0.915(0.849,0.985) | 0.019 | 0.918(0.852,0.990) | 0.026 | 0.924(0.856,0.997) | 0.042 |
| Q4 | 0.863(0.802,0.929) | ＜0.001 | 0.875(0.810,0.944) | 0.001 | 0.885(0.818,0.958) | 0.003 |
| *P* for trend | ＜0.001 | | ＜0.001 | | ＜0.001 | |
| **3-year cumulative TyG** | | | | | | |
| Per SD | 0.974(0.950,0.998) | 0.037 | 0.975(0.951,1.000) | 0.049 | 0.981(0.956,1.007) | 0.150 |
| Q1 | Reference | | Reference | | Reference | |
| Q2 | 1.026(0.953,1.106) | 0.496 | 1.030(0.956,1.109) | 0.438 | 1.029(0.956,1.107) | 0.450 |
| Q3 | 0.959(0.889,1.036) | 0.287 | 0.963(0.893,1.039) | 0.329 | 0.962(0.892,1.037) | 0.311 |
| Q4 | 0.921(0.857,0.989) | 0.024 | 0.925(0.860,0.994) | 0.033 | 0.933(0.867,1.004) | 0.063 |
| *P* for trend | 0.005 | | 0.008 | | 0.020 | |
| **3-year cumulative TyG-BMI** | | | | | | |
| Per SD | 0.957(0.933,0.981) | 0.001 | 0.957(0.933,0.982) | 0.001 | 0.964(0.939,0.990) | 0.006 |
| Q1 | Reference | | Reference | | Reference | |
| Q2 | 0.994(0.922,1.073) | 0.886 | 0.993(0.920,1.071) | 0.846 | 0.998(0.926,1.077) | 0.967 |
| Q3 | 0.919(0.851,0.993) | 0.033 | 0.917(0.849,0.991) | 0.029 | 0.929(0.860,1.004) | 0.062 |
| Q4 | 0.893(0.830,0.962) | 0.003 | 0.896(0.832,0.965) | 0.004 | 0.912(0.846,0.983) | 0.016 |
| *P* for trend | ＜0.001 | | 0.001 | | 0.004 | |
| **3-year cumulative TyG-WC** | | | | | | |
| Per SD | 0.934(0.911,0.958) | ＜0.001 | 0.933(0.909,0.956) | ＜0.001 | 0.938(0.914,0.963) | ＜0.001 |
| Q1 | Reference | | Reference | | Reference | |
| Q2 | 1.005(0.929,1.088) | 0.899 | 0.999(0.924,1.080) | 0.970 | 1.000(0.926,1.081) | 0.994 |
| Q3 | 0.930(0.863,1.002) | 0.057 | 0.925(0.859,0.996) | 0.039 | 0.932(0.865,1.005) | 0.066 |
| Q4 | 0.850(0.790,0.914) | ＜0.001 | 0.846(0.787,0.909) | ＜0.001 | 0.858(0.797,0.924) | ＜0.001 |
| *P* for trend | ＜0.001 | | ＜0.001 | | ＜0.001 | |
| **3-year cumulative TyG-WHtR** | | | | | | |
| Per SD | 0.937(0.914,0.962) | 0.000 | 0.941(0.917,0.967) | 0.000 | 0.947(0.921,0.974) | 0.000 |
| Q1 | Reference | | Reference | | Reference | |
| Q2 | 0.948(0.879,1.023) | 0.166 | 0.948(0.879,1.023) | 0.171 | 0.955(0.885,1.029) | 0.226 |
| Q3 | 0.884(0.816,0.959) | 0.003 | 0.889(0.819,0.965) | 0.005 | 0.895(0.824,0.972) | 0.009 |
| Q4 | 0.839(0.779,0.904) | ＜0.001 | 0.848(0.785,0.917) | ＜0.001 | 0.863(0.797,0.935) | ＜0.001 |
| *P* for trend | ＜0.001 | | ＜0.001 | | ＜0.001 | |
| **3-year mean change TyG** | | | | | | |
| Per SD | 0.994(0.967,1.022) | 0.669 | 0.995(0.967,1.022) | 0.698 | 0.998(0.970,1.027) | 0.907 |
| Q1 | Reference | | Reference | | Reference | |
| Q2 | 0.969(0.900,1.043) | 0.399 | 0.971(0.903,1.044) | 0.429 | 0.975(0.906,1.049) | 0.496 |
| Q3 | 0.961(0.888,1.041) | 0.326 | 0.964(0.892,1.042) | 0.352 | 0.969(0.897,1.046) | 0.418 |
| Q4 | 0.997(0.922,1.077) | 0.937 | 0.998(0.924,1.079) | 0.965 | 1.007(0.931,1.089) | 0.864 |
| P for trend | 0.727 | | 0.768 | | 0.854 | |
| **3-year mean change TyG-BMI** | | | | | | |
| Per SD | 0.976(0.951,1.002) | 0.067 | 0.973(0.948,0.998) | 0.038 | 0.975(0.950,1.001) | 0.061 |
| Q1 | Reference | | Reference | | Reference | |
| Q2 | 0.925(0.858,0.998) | 0.045 | 0.926(0.859,0.999) | 0.046 | 0.932(0.865,1.005) | 0.066 |
| Q3 | 0.900(0.831,0.975) | 0.011 | 0.897(0.828,0.972) | 0.008 | 0.905(0.836,0.980) | 0.015 |
| Q4 | 0.952(0.884,1.026) | 0.196 | 0.947(0.879,1.019) | 0.146 | 0.953(0.883,1.027) | 0.208 |
| P for trend | 0.178 | | 0.121 | | 0.175 | |
| **3-year mean change TyG-WC** | | | | | | |
| Per SD | 1.002(0.977,1.028) | 0.874 | 1.001(0.976,1.027) | 0.954 | 1.002(0.976,1.028) | 0.882 |
| Q1 | Reference | | Reference | | Reference | |
| Q2 | 1.010(0.932,1.095) | 0.801 | 1.006(0.928,1.089) | 0.890 | 1.011(0.934,1.095) | 0.788 |
| Q3 | 1.057(0.979,1.141) | 0.155 | 1.049(0.973,1.132) | 0.214 | 1.051(0.975,1.133) | 0.191 |
| Q4 | 1.024(0.952,1.103) | 0.520 | 1.021(0.949,1.099) | 0.571 | 1.025(0.952,1.104) | 0.516 |
| P for trend | 0.339 | | 0.376 | | 0.347 | |
| **3-year mean change TyG-WHtR** | | | | | | |
| Per SD | 1.002(0.977,1.028) | 0.859 | 1.001(0.976,1.027) | 0.926 | 1.002(0.977,1.028) | 0.859 |
| Q1 | Reference | | Reference | | Reference | |
| Q2 | 1.010(0.931,1.095) | 0.816 | 1.003(0.926,1.088) | 0.932 | 1.009(0.931,1.093) | 0.830 |
| Q3 | 1.053(0.975,1.136) | 0.189 | 1.042(0.966,1.125) | 0.282 | 1.045(0.970,1.127) | 0.243 |
| Q4 | 1.025(0.952,1.103) | 0.510 | 1.023(0.951,1.100) | 0.542 | 1.026(0.953,1.105) | 0.495 |
| P for trend | 0.299 | | 0.321 | | 0.295 | |

CART,classification and regression trees; other abbreviations as in Table 1; TR, time ratio; CI, confidence interval; SD, standard deviations. Model I was unadjusted; Model II was adjusted for age, gender, smoking status and drinking status; Model III was adjusted for age, gender, smoking status, drinking status, SBP, diabetes, hypertension and LDL-C.

**TableS6.** Time ratio of associations between baseline, cumulative and annual mean change TyG-related indices with cardiovascular events among elderly rural residents with CKM syndrome stages 0–3（original non-imputed data）

| **Exposures** | **Model 1** | | **Model 2** | | **Model 3** | |
| --- | --- | --- | --- | --- | --- | --- |
|  | **TR(95%CI)** | ***P* value** | **TR(95%CI)** | ***P* value** | **TR(95%CI)** | ***P* value** |
| **Baseline TyG** | | | | | | |
| Per SD | 0.997 (0.971-1.024) | 0.830 | 1.000 (0.973-1.027) | 0.980 | 0.999 (0.971-1.027) | 0.923 |
| Q1 | Reference | | Reference | | Reference | |
| Q2 | 1.000 (0.927-1.078) | 0.994 | 0.996 (0.924-1.073) | 0.911 | 1.000 (0.928-1.078) | 0.996 |
| Q3 | 1.017 (0.943-1.098) | 0.658 | 1.020 (0.946-1.100) | 0.603 | 1.007 (0.933-1.087) | 0.858 |
| Q4 | 0.964 (0.895-1.040) | 0.344 | 0.969 (0.900-1.044) | 0.413 | 0.967 (0.895-1.045) | 0.393 |
| *P* for trend | 0.444 | | 0.556 | | 0.457 | |
| **Baseline TyG-BMI** | | | | | | |
| Per SD | 0.992 (0.966-1.019) | 0.569 | 0.998 (0.971-1.025) | 0.870 | 1.004 (0.976-1.032) | 0.803 |
| Q1 | Reference | | Reference | | Reference | |
| Q2 | 1.065 (0.988-1.148) | 0.102 | 1.066 (0.989-1.149) | 0.096 | 1.062 (0.985-1.145) | 0.119 |
| Q3 | 1.051 (0.974-1.133) | 0.199 | 1.056 (0.980-1.139) | 0.154 | 1.067 (0.988-1.152) | 0.101 |
| Q4 | 1.019 (0.946-1.098) | 0.612 | 1.034 (0.960-1.115) | 0.377 | 1.050 (0.971-1.135) | 0.225 |
| *P* for trend | 0.703 | | 0.444 | | 0.240 | |
| **Baseline TyG-WC** | | | | | | |
| Per SD | 0.954 (0.929-0.979) | <0.001 | 0.956 (0.931-0.981) | 0.001 | 0.957 (0.931-0.984) | 0.002 |
| Q1 | Reference | | Reference | | Reference | |
| Q2 | 1.068 (0.989-1.154) | 0.095 | 1.060 (0.982-1.145) | 0.135 | 1.070 (0.990-1.155) | 0.087 |
| Q3 | 1.017 (0.942-1.097) | 0.668 | 1.015 (0.942-1.095) | 0.694 | 1.010 (0.935-1.090) | 0.806 |
| Q4 | 0.903 (0.839-0.972) | 0.007 | 0.907 (0.842-0.976) | 0.009 | 0.914 (0.846-0.987) | 0.022 |
| *P* for trend | 0.002 | | 0.004 | | 0.010 | |
| **Baseline TyG-WHtR** | | | | | | |
| Per SD | 0.953 (0.929-0.979) | <0.001 | 0.961 (0.935-0.988) | 0.005 | 0.963 (0.936-0.992) | 0.012 |
| Q1 | Reference | | Reference | | Reference | |
| Q2 | 0.998 (0.924-1.077) | 0.952 | 1.001 (0.927-1.080) | 0.986 | 0.998 (0.924-1.077) | 0.951 |
| Q3 | 1.001 (0.927-1.081) | 0.984 | 1.008 (0.933-1.089) | 0.841 | 1.002 (0.926-1.085) | 0.957 |
| Q4 | 0.865 (0.803-0.932) | <0.001 | 0.882 (0.817-0.952) | 0.001 | 0.882 (0.814-0.955) | 0.002 |
| *P* for trend | <0.001 | | 0.002 | | 0.003 | |
| **2-year cumulative TyG** | | | | | | |
| Per SD | 0.985 (0.958-1.014) | 0.306 | 0.987 (0.959-1.015) | 0.352 | 0.990 (0.961-1.019) | 0.489 |
| Q1 | Reference | | Reference | | Reference | |
| Q2 | 0.974 (0.898-1.057) | 0.531 | 0.975 (0.899-1.057) | 0.540 | 0.971 (0.895-1.053) | 0.474 |
| Q3 | 0.998 (0.920-1.083) | 0.964 | 0.996 (0.918-1.080) | 0.917 | 0.990 (0.912-1.075) | 0.815 |
| Q4 | 0.933 (0.861-1.011) | 0.091 | 0.938 (0.865-1.016) | 0.116 | 0.944 (0.869-1.026) | 0.173 |
| *P* for trend | 0.150 | | 0.178 | | 0.252 | |
| **2-year cumulative TyG-BMI** | | | | | | |
| Per SD | 0.980 (0.953-1.009) | 0.170 | 0.982 (0.954-1.011) | 0.231 | 0.990 (0.961-1.020) | 0.499 |
| Q1 | Reference | | Reference | | Reference | |
| Q2 | 1.000 (0.922-1.084) | 0.992 | 0.999 (0.921-1.083) | 0.981 | 1.017 (0.938-1.103) | 0.679 |
| Q3 | 1.002 (0.923-1.086) | 0.969 | 1.003 (0.925-1.088) | 0.940 | 1.022 (0.942-1.110) | 0.599 |
| Q4 | 0.970 (0.895-1.052) | 0.461 | 0.978 (0.902-1.061) | 0.596 | 1.001 (0.921-1.089) | 0.973 |
| *P* for trend | 0.489 | | 0.633 | | 0.948 | |
| **2-year cumulative TyG-WC** | | | | | | |
| Per SD | 0.942 (0.915-0.969) | <0.001 | 0.942 (0.915-0.969) | <0.001 | 0.946 (0.919-0.975) | <0.001 |
| Q1 | Reference | | Reference | | Reference | |
| Q2 | 0.982 (0.903-1.067) | 0.663 | 0.976 (0.898-1.061) | 0.564 | 0.984 (0.906-1.070) | 0.711 |
| Q3 | 0.918 (0.845-0.996) | 0.040 | 0.918 (0.846-0.996) | 0.039 | 0.924 (0.851-1.003) | 0.060 |
| Q4 | 0.855 (0.789-0.927) | <0.001 | 0.855 (0.789-0.927) | <0.001 | 0.869 (0.800-0.944) | 0.001 |
| *P* for trend | <0.001 | | <0.001 | | <0.001 | |
| **2-year cumulative TyG-WHtR** | | | | | | |
| Per SD | 0.944 (0.918-0.971) | <0.001 | 0.949 (0.921-0.978) | 0.001 | 0.954 (0.924-0.984) | 0.003 |
| Q1 | Reference | | Reference | | Reference | |
| Q2 | 1.000 (0.920-1.087) | 0.993 | 1.004 (0.924-1.091) | 0.923 | 1.017 (0.936-1.105) | 0.693 |
| Q3 | 0.929 (0.856-1.008) | 0.078 | 0.935 (0.861-1.015) | 0.108 | 0.942 (0.867-1.023) | 0.157 |
| Q4 | 0.879 (0.811-0.953) | 0.002 | 0.895 (0.823-0.973) | 0.009 | 0.911 (0.835-0.993) | 0.034 |
| *P* for trend | <0.001 | | 0.003 | | 0.011 | |
| **3-year cumulative TyG** | | | | | | |
| Per SD | 0.994 (0.959-1.030) | 0.745 | 0.997 (0.961-1.033) | 0.850 | 1.003 (0.967-1.041) | 0.857 |
| Q1 | Reference | | Reference | | Reference | |
| Q2 | 0.996 (0.899-1.103) | 0.932 | 0.998 (0.901-1.106) | 0.972 | 1.010 (0.912-1.118) | 0.854 |
| Q3 | 0.970 (0.876-1.073) | 0.553 | 0.980 (0.885-1.085) | 0.702 | 0.984 (0.888-1.090) | 0.754 |
| Q4 | 0.962 (0.870-1.064) | 0.453 | 0.966 (0.872-1.070) | 0.505 | 0.978 (0.881-1.086) | 0.679 |
| *P* for trend | 0.383 | | 0.458 | | 0.587 | |
| **3-year cumulative TyG-BMI** | | | | | | |
| Per SD | 0.952 (0.918-0.987) | 0.007 | 0.952 (0.918-0.987) | 0.008 | 0.962 (0.927-0.998) | 0.041 |
| Q1 | Reference | | Reference | | Reference | |
| Q2 | 0.944 (0.850-1.047) | 0.273 | 0.942 (0.849-1.045) | 0.256 | 0.947 (0.854-1.050) | 0.301 |
| Q3 | 0.866 (0.782-0.959) | 0.006 | 0.868 (0.783-0.961) | 0.006 | 0.883 (0.798-0.978) | 0.017 |
| Q4 | 0.888 (0.801-0.984) | 0.023 | 0.891 (0.804-0.987) | 0.028 | 0.917 (0.826-1.018) | 0.104 |
| *P* for trend | 0.008 | | 0.010 | | 0.051 | |
| **3-year cumulative TyG-WC** | | | | | | |
| Per SD | 0.943 (0.910-0.977) | 0.001 | 0.945 (0.912-0.979) | 0.002 | 0.954 (0.920-0.990) | 0.012 |
| Q1 | Reference | | Reference | | Reference | |
| Q2 | 0.948 (0.854-1.051) | 0.311 | 0.944 (0.852-1.047) | 0.275 | 0.932 (0.841-1.032) | 0.175 |
| Q3 | 0.896 (0.809-0.993) | 0.036 | 0.897 (0.810-0.993) | 0.036 | 0.900 (0.812-0.997) | 0.043 |
| Q4 | 0.870 (0.786-0.963) | 0.007 | 0.877 (0.793-0.971) | 0.011 | 0.890 (0.802-0.987) | 0.027 |
| *P* for trend | 0.004 | | 0.007 | | 0.022 | |
| **3-year cumulative TyG-WHtR** | | | | | | |
| Per SD | 0.933 (0.901-0.968) | <0.001 | 0.940 (0.906-0.976) | 0.001 | 0.949 (0.913-0.986) | 0.008 |
| Q1 | Reference | | Reference | | Reference | |
| Q2 | 0.967 (0.872-1.074) | 0.533 | 0.976 (0.879-1.083) | 0.642 | 0.988 (0.890-1.096) | 0.816 |
| Q3 | 0.890 (0.804-0.986) | 0.026 | 0.902 (0.814-1.000) | 0.050 | 0.908 (0.819-1.007) | 0.067 |
| Q4 | 0.858 (0.775-0.950) | 0.003 | 0.879 (0.791-0.977) | 0.016 | 0.903 (0.811-1.006) | 0.064 |
| *P* for trend | 0.001 | | 0.006 | | 0.024 | |
| **3-year mean change TyG** | | | | | | |
| Per SD | 1.023 (0.987-1.060) | 0.214 | 1.021 (0.986-1.058) | 0.242 | 1.027 (0.991-1.065) | 0.141 |
| Q1 | Reference | | Reference | | Reference | |
| Q2 | 0.968 (0.878-1.068) | 0.519 | 0.973 (0.884-1.072) | 0.586 | 1.005 (0.912-1.108) | 0.917 |
| Q3 | 0.966 (0.876-1.065) | 0.491 | 0.966 (0.877-1.064) | 0.478 | 0.981 (0.890-1.081) | 0.697 |
| Q4 | 1.033 (0.936-1.141) | 0.520 | 1.031 (0.934-1.137) | 0.545 | 1.056 (0.955-1.167) | 0.290 |
| *P* for trend | 0.599 | | 0.611 | | 0.411 | |
| **3-year mean change TyG-BMI** | | | | | | |
| Per SD | 0.989 (0.955-1.024) | 0.535 | 0.982 (0.948-1.017) | 0.302 | 0.985 (0.951-1.021) | 0.405 |
| Q1 | Reference | | Reference | | Reference | |
| Q2 | 0.959 (0.869-1.059) | 0.407 | 0.958 (0.869-1.057) | 0.395 | 0.976 (0.885-1.077) | 0.633 |
| Q3 | 0.930 (0.843-1.025) | 0.144 | 0.919 (0.834-1.013) | 0.089 | 0.930 (0.843-1.025) | 0.144 |
| Q4 | 0.958 (0.868-1.057) | 0.394 | 0.945 (0.857-1.042) | 0.253 | 0.953 (0.862-1.052) | 0.338 |
| *P* for trend | 0.315 | | 0.176 | | 0.218 | |
| **3-year mean change TyG-WC** | | | | | | |
| Per SD | 1.020 (0.985-1.056) | 0.272 | 1.016 (0.981-1.052) | 0.372 | 1.015 (0.980-1.052) | 0.405 |
| Q1 | Reference | | Reference | | Reference | |
| Q2 | 1.024 (0.929-1.129) | 0.636 | 1.012 (0.919-1.115) | 0.803 | 1.016 (0.922-1.119) | 0.754 |
| Q3 | 1.003 (0.910-1.105) | 0.957 | 0.990 (0.899-1.090) | 0.835 | 0.988 (0.897-1.088) | 0.805 |
| Q4 | 1.066 (0.966-1.176) | 0.204 | 1.057 (0.958-1.165) | 0.268 | 1.054 (0.953-1.165) | 0.304 |
| *P* for trend | 0.288 | | 0.371 | | 0.443 | |
| **3-year mean change TyG-WHtR** | | | | | | |
| Per SD | 1.020 (0.985-1.057) | 0.259 | 1.017 (0.982-1.053) | 0.348 | 1.016 (0.981-1.053) | 0.379 |
| Q1 | Reference | | Reference | | Reference | |
| Q2 | 1.020 (0.925-1.124) | 0.695 | 1.008 (0.915-1.111) | 0.873 | 1.014 (0.920-1.118) | 0.781 |
| Q3 | 1.008 (0.914-1.111) | 0.878 | 0.994 (0.903-1.095) | 0.905 | 0.993 (0.902-1.094) | 0.889 |
| Q4 | 1.056 (0.957-1.165) | 0.280 | 1.047 (0.950-1.154) | 0.356 | 1.045 (0.946-1.155) | 0.385 |
| *P* for trend | 0.345 | | 0.436 | | 0.503 | |

TR, time ratio; CI, confidence interval; SD, standard deviations; other abbreviations as in Table 1. Model I was unadjusted; Model II was adjusted for age, gender, smoking status and drinking status; Model III was adjusted for age, gender, smoking status, drinking status, SBP, diabetes, hypertension and LDL-C.

**TableS7.** Predictive value of cardiovascular events prediction model(original non-imputed data)

| **Model** | **C-index(95%CI）** | **4-year AUC(95%CI）** | **5-year AUC(95%CI）** | 6-**Year AUC(95%CI）** |
| --- | --- | --- | --- | --- |
| Baseline TyG | 0.606(0.586-0.629) | 0.606(0.575-0.637) | 0.628(0.601-0.654) | 0.682(0.631-0.732) |
| Baseline TyG-BMI | 0.608(0.587-0.633) | 0.609(0.578-0.640) | 0.626(0.600-0.652) | 0.671(0.608-0.734) |
| Baseline TyG-WC | 0.613(0.589-0.636) | 0.612(0.581-0.643) | 0.637(0.601-0.673) | 0.646(0.527-0.766) |
| Baseline TyG-WHtR | 0.611(0.588-0.632) | 0.611(0.581-0.641) | 0.628(0.599-0.656) | 0.626(0.571-0.680) |
| 2-year cumulative TyG | 0.609(0.585-0.630) | 0.602(0.571-0.632) | 0.629(0.597-0.660) | 0.689(0.637-0.741) |
| 2-year cumulative TyG-BMI | 0.607(0.583-0.630) | 0.604(0.575-0.632) | 0.636(0.603-0.670) | 0.652(0.529-0.775) |
| 2-year cumulative TyG-WC | 0.614(0.594-0.634) | 0.609(0.577-0.641) | 0.632(0.600-0.663) | 0.649(0.528-0.770) |
| 2-year cumulative TyG-WHtR | 0.612(0.589-0.633) | 0.607(0.571-0.642) | 0.631(0.598-0.665) | 0.650(0.545-0.755) |
| 3-year cumulative TyG | 0.602(0.579-0.629) | 0.604(0.566-0.642) | 0.628(0.599-0.656) | 0.676(0.616-0.736) |
| 3-year cumulative TyG-BMI | 0.605(0.576-0.625) | 0.603(0.566-0.639) | 0.631(0.600-0.662) | 0.645(0.533-0.757) |
| 3-year cumulative TyG-WC | 0.607(0.585-0.637) | 0.609(0.571-0.647) | 0.630(0.600-0.660) | 0.640(0.524-0.756) |
| 3-year cumulative TyG-WHtR | 0.609(0.584-0.633) | 0.610(0.570-0.649) | 0.624(0.598-0.651) | 0.659(0.593-0.724) |
| 3-year mean change TyG | 0.605(0.580-0.631) | 0.607(0.572-0.642) | 0.627(0.601-0.653) | 0.635(0.569-0.701) |
| 3-year mean change TyG-BMI | 0.603(0.577-0.629) | 0.607(0.576-0.639) | 0.631(0.596-0.666) | 0.625(0.498-0.752) |
| 3-year mean change TyG-WC | 0.605(0.582-0.624) | 0.608(0.576-0.640) | 0.635(0.603-0.666) | 0.636(0.516-0.756) |
| 3-year mean change TyG-WHtR | 0.603(0.579-0.628) | 0.608(0.570-0.646) | 0.624(0.596-0.653) | 0.658(0.602-0.714) |

AUC, area under the curve; CI, confidence interval; other abbreviations as in Table 1.

**TableS8.** Comparison and incremental predictive value of cardiovascular events prediction model(data imputed with CART)

| **Model variables** | **C-index** | **95%CI** | **∆ AIC** | **∆ BIC** | **LRT *P*-value** |
| --- | --- | --- | --- | --- | --- |
| base model(basic covariates) | 0.590 | 0.494-0.687 | 0.0 | 0.0 | Ref |
| Basic + 3-year cumulative TyG-WC | 0.601 | 0.505-0.697 | -32.5 | -27.8 | ＜0.001 |
| Basic + 2-year cumulative TyG-WC | 0.600 | 0.504-0.696 | -30.7 | -26.0 | ＜0.001 |
| Basic + basline TyG-WC | 0.597 | 0.501-0.693 | -25.3 | -20.6 | ＜0.001 |
| Basic + 3-year cumulative TyG-WHtR | 0.596 | 0.500-0.693 | -21.1 | -16.4 | ＜0.001 |
| Basic + 2-year cumulative TyG-WHtR | 0.596 | 0.499-0.692 | -20.0 | -15.3 | ＜0.001 |
| Basic + basline TyG-WHtR | 0.594 | 0.497-0.690 | -16.8 | -12.1 | ＜0.001 |
| Basic + 3-year cumulative TyG-BMI | 0.594 | 0.497-0.690 | -9.8 | -5.0 | ＜0.001 |
| Basic + 2-year cumulative TyG-BMI | 0.592 | 0.496-0.689 | -7.5 | -2.8 | 0.002 |
| Basic + 2-year cumulative TyG | 0.592 | 0.496-0.688 | -4.6 | 0.1 | 0.011 |
| Basic + 3-year cumulative TyG | 0.592 | 0.495-0.688 | -4.6 | 0.1 | 0.011 |
| Basic + basline TyG-BMI | 0.590 | 0.494-0.687 | -3.1 | 1.6 | 0.021 |
| Basic + 3-year mean change TyG-BMI | 0.593 | 0.496-0.689 | -1.3 | 3.4 | 0.078 |
| Basic + basline TyG | 0.590 | 0.493-0.686 | -0.3 | 4.4 | 0.115 |
| Basic + 3-year mean change TyG | 0.590 | 0.494-0.687 | 1.9 | 6.6 | 0.568 |
| Basic + 3-year mean change TyG-WHtR | 0.590 | 0.494-0.687 | 1.7 | 6.4 | 0.609 |
| Basic + 3-year mean change TyG-WC | 0.590 | 0.494-0.687 | 1.7 | 6.5 | 0.635 |

CART,classification and regression trees; AUC, area under the curve; ΔAIC, difference in AIC compared to base model; ΔBIC, difference in BIC compared to base model; LRT, likelihood ratio test; other abbreviations as in Table 1; base model include age, gender, smoking status, drinking status, SBP, diabetes, hypertension and LDL-C.

**TableS9.** Comparison and incremental predictive value of cardiovascular events prediction model(original non-imputed data)

| **Model variables** | **C-index** | **95%CI** | **∆ AIC** | **∆ BIC** | **LRT *P*-value** |
| --- | --- | --- | --- | --- | --- |
| base model(basic covariates) | 0.603 | 0.579-0.623 | 0.0 | 0.0 | Ref |
| Basic + 2-year cumulative TyG-WHtR | 0.612 | 0.589-0.633 | -10.4 | -6.2 | 0.007 |
| Basic + 3-year cumulative TyG-WHtR | 0.609 | 0.584-0.633 | -9.0 | -4.9 | 0.007 |
| Basic + 2-year cumulative TyG-WC | 0.614 | 0.594-0.634 | -9.0 | -4.9 | 0.007 |
| Basic + 3-year cumulative TyG-WC | 0.607 | 0.585-0.637 | -7.9 | -3.8 | 0.010 |
| Basic + baseline TyG-WHtR | 0.611 | 0.588-0.632 | -5.8 | -1.6 | 0.018 |
| Basic + baseline TyG-WC | 0.613 | 0.589-0.636 | -4.9 | -0.8 | 0.025 |
| Basic + 3-year cumulative TyG-BMI | 0.605 | 0.576-0.625 | -4.1 | 0.0 | 0.032 |
| Basic + 2-year cumulative TyG-BMI | 0.607 | 0.583-0.630 | -3.0 | 1.2 | 0.056 |
| Basic + baseline TyG-BMI | 0.608 | 0.587-0.633 | -0.6 | 3.5 | 0.208 |
| Basic + 3-year mean change TyG | 0.605 | 0.580-0.631 | 0.5 | 4.6 | 0.406 |
| Basic + 3-year mean change TyG-BMI | 0.603 | 0.577-0.629 | 1.2 | 5.3 | 0.582 |
| Basic + 3-year mean change TyG-WHtR | 0.603 | 0.579-0.628 | 1.4 | 5.5 | 0.642 |
| Basic + baseline TyG | 0.606 | 0.586-0.629 | 1.5 | 5.6 | 0.654 |
| Basic + 3-year mean change TyG-WC | 0.605 | 0.582-0.624 | 1.5 | 5.7 | 0.654 |
| Basic + 2-year cumulative TyG | 0.609 | 0.585-0.630 | 1.6 | 5.8 | 0.680 |
| Basic + 3-year cumulative TyG | 0.602 | 0.579-0.629 | 2.0 | 6.1 | 0.924 |

AUC, area under the curve; ΔAIC, difference in AIC compared to base model; ΔBIC, difference in BIC compared to base model; LRT, likelihood ratio test; other abbreviations as in Table 1; base model include age, gender, smoking status, drinking status, SBP, diabetes, hypertension and LDL-C.

**TableS10.** Pairwise comparison of cardiovascular disease prediction models based on **baseline** TyG-related indices(data imputed with PMM)

| **Model Pairs** | **ΔAUC** | **Lower95%CI** | **Upper95%CI** | ***P*-value** | **Adjusted *P*-value** |
| --- | --- | --- | --- | --- | --- |
| base model vs basline TyG model | -0.039 | -0.054 | -0.023 | ＜0.001 | **＜0.001** |
| base model vs basline TyG-BMI model | -0.029 | -0.045 | -0.014 | ＜0.001 | **＜0.001** |
| base model vs basline TyG-WC model | -0.072 | -0.104 | -0.041 | ＜0.001 | **＜0.001** |
| base model vs basline TyG-WHtR model | -0.061 | -0.088 | -0.034 | ＜0.001 | **＜0.001** |
| basline TyG model vs basline TyG-BMI model | 0.009 | -0.004 | 0.023 | 0.172 | 0.172 |
| basline TyG model vs basline TyG-WC model | -0.034 | -0.059 | -0.009 | 0.008 | **0.012** |
| basline TyG model vs basline TyG-WHtR model | -0.023 | -0.044 | -0.002 | 0.035 | **0.038** |
| basline TyG-BMI model vs basline TyG-WC model | -0.043 | -0.066 | -0.020 | ＜0.001 | **＜0.001** |
| basline TyG-BMI model vs basline TyG-WHtR model | -0.032 | -0.05 | -0.014 | ＜0.001 | **＜0.001** |
| basline TyG-WC model vs basline TyG-WHtR model | 0.011 | 0.002 | 0.021 | 0.021 | **0.026** |

PMM, predictive mean matching; AUC, area under the curve; other abbreviations as in Table 1. ΔAUC: the difference between the two models; adjusted P-value: P values adjusted for multiple testing using Benjamini-Hochberg procedures, respectively.

**TableS11.** Pairwise comparison of cardiovascular disease prediction models based on **2-year cumulative** TyG-related indices(data imputed with PMM)

| **Model Pairs** | **ΔAUC** | **Lower95%CI** | **Upper95%CI** | ***P*-value** | **Adjusted *P*-value** |
| --- | --- | --- | --- | --- | --- |
| base model vs 2-year cumulative TyG model | -0.047 | -0.067 | -0.028 | ＜0.001 | **＜0.001** |
| base model vs 2-year cumulative TyG-BMI model | -0.033 | -0.053 | -0.014 | ＜0.001 | **＜0.001** |
| base model vs 2-year cumulative TyG-WC model | -0.071 | -0.105 | -0.038 | ＜0.001 | **＜0.001** |
| base model vs 2-year cumulative TyG-WHtR model | -0.059 | -0.087 | -0.03 | ＜0.001 | **＜0.001** |
| 2-year cumulative TyG model vs 2-year cumulative TyG-BMI model | 0.014 | -0.004 | 0.031 | 0.127 | 0.141 |
| 2-year cumulative TyG model vs 2-year cumulative TyG-WC model | -0.024 | -0.051 | 0.002 | 0.070 | 0.087 |
| 2-year cumulative TyG model vs 2-year cumulative TyG-WHtR model | -0.012 | -0.034 | 0.011 | 0.315 | 0.315 |
| 2-year cumulative TyG-BMI model vs 2-year cumulative TyG-WC model | -0.038 | -0.06 | -0.016 | ＜0.001 | **＜0.001** |
| 2-year cumulative TyG-BMI model vs 2-year cumulative TyG-WHtR model | -0.025 | -0.042 | -0.009 | ＜0.001 | **＜0.001** |
| 2-year cumulative TyG-WC model vs 2-year cumulative TyG-WHtR model | 0.013 | 0.002 | 0.024 | 0.026 | **0.037** |

PMM, predictive mean matching; AUC, area under the curve; other abbreviations as in Table 1.ΔAUC: the difference between the two models; adjusted P-value: P values adjusted for multiple testing using Benjamini-Hochberg procedures, respectively.

**TableS12**. Pairwise comparison of cardiovascular disease prediction models based on **3-year cumulative** TyG-related indices(data imputed with PMM)

| **Model Pairs** | **ΔAUC** | **Lower95%CI** | **Upper95%CI** | ***P*-value** | **Adjusted *P*-value** |
| --- | --- | --- | --- | --- | --- |
| base model vs 3-year cumulative TyG model | -0.037 | -0.055 | -0.019 | ＜0.001 | **＜0.001** |
| base model vs 3-year cumulative TyG-BMI model | -0.036 | -0.058 | -0.015 | ＜0.001 | **＜0.001** |
| base model vs 3-year cumulative TyG-WC model | -0.067 | -0.099 | -0.034 | ＜0.001 | **＜0.001** |
| base model vs 3-year cumulative TyG-WHtR model | -0.054 | -0.082 | -0.027 | ＜0.001 | **＜0.001** |
| 3-year cumulative TyG model vs 3-year cumulative TyG-BMI model | 0.001 | -0.018 | 0.020 | 0.915 | 0.915 |
| 3-year cumulative TyG model vs 3-year cumulative TyG-WC model | -0.029 | -0.055 | -0.003 | 0.028 | **0.040** |
| 3-year cumulative TyG model vs 3-year cumulative TyG-WHtR model | -0.017 | -0.039 | 0.005 | 0.134 | 0.149 |
| 3-year cumulative TyG-BMI model vs 3-year cumulative TyG-WC model | -0.030 | -0.050 | -0.010 | 0.003 | **0.006** |
| 3-year cumulative TyG-BMI model vs 3-year cumulative TyG-WHtR model | -0.018 | -0.032 | -0.003 | 0.017 | **0.028** |
| 3-year cumulative TyG-WC model vs 3-year cumulative TyG-WHtR model | 0.012 | 0.001 | 0.024 | 0.035 | **0.044** |

PMM, predictive mean matching; AUC, area under the curve; other abbreviations as in Table 1. ΔAUC: the difference between the two models; adjusted P-value: P values adjusted for multiple testing using Benjamini-Hochberg procedures, respectively.

**TableS13.** Pairwise comparison of cardiovascular disease prediction models based on **3-year mean change** TyG-related indices(data imputed with PMM)

| **Model Pairs** | **ΔAUC** | **Lower95%CI** | **Upper95%CI** | ***P*-value** | **Adjusted *P*-value** |
| --- | --- | --- | --- | --- | --- |
| base model vs 3-year mean change TyG | -0.007 | -0.014 | 0.000 | 0.046 | 0.339 |
| base model vs 3-year mean change TyG-BMI | 0.008 | -0.006 | 0.021 | 0.264 | 0.413 |
| base model vs 3-year mean change TyG-WC | -0.003 | -0.012 | 0.006 | 0.484 | 0.484 |
| base model vs 3-year mean change TyG-WHtR | -0.003 | -0.012 | 0.006 | 0.406 | 0.451 |
| 3-year mean change TyG vs 3-year mean change TyG-BMI | 0.015 | -0.001 | 0.031 | 0.068 | 0.339 |
| 3-year mean change TyG vs 3-year mean change TyG-WC | 0.004 | -0.004 | 0.012 | 0.2402 | 0.413 |
| 3-year mean change TyG vs 3-year mean change TyG-WHtR | 0.004 | -0.004 | 0.012 | 0.289 | 0.413 |
| 3-year mean change TyG-BMI vs 3-year mean change TyG-WC | -0.011 | -0.026 | 0.005 | 0.171 | 0.413 |
| 3-year mean change TyG-BMI vs 3-year mean change TyG-WHtR | -0.011 | -0.026 | 0.004 | 0.158 | 0.413 |
| 3-year mean change TyG-WC vs 3-year mean change TyG-WHtR | 0.000 | -0.001 | 0.001 | 0.334 | 0.418 |

PMM, predictive mean matching; AUC, area under the curve; other abbreviations as in Table 1. ΔAUC: the difference between the two models; adjusted P-value: P values adjusted for multiple testing using Benjamini-Hochberg procedures, respectively.

**TableS14.** Pairwise comparison of cardiovascular disease prediction models based on **TyG** indices(data imputed with PMM)

| **Model Pairs** | **ΔAUC** | **Lower95%CI** | **Upper95%CI** | ***P*-value** | **Adjusted *P*-value** |
| --- | --- | --- | --- | --- | --- |
| base model vs basline TyG model | -0.039 | -0.054 | -0.023 | ＜0.001 | **＜0.001** |
| base model vs 2-year cumulative TyG model | -0.047 | -0.067 | -0.027 | ＜0.001 | **＜0.001** |
| base model vs 3-year cumulative TyG model | -0.037 | -0.056 | -0.019 | ＜0.001 | **＜0.001** |
| base model vs 3-year mean change TyG model | -0.007 | -0.014 | 0.000 | 0.047 | 0.062 |
| basline TyG vs 2-year cumulative TyG model | -0.008 | -0.018 | 0.001 | 0.0809 | 0.100 |
| basline TyG vs 3-year cumulative TyG model | 0.001 | -0.011 | 0.014 | 0.8345 | 0.835 |
| basline TyG vs 3-year mean change TyG model | 0.032 | 0.019 | 0.044 | ＜0.001 | **＜0.001** |
| **2-year cumulative TyG vs 3-year cumulative TyG model** | **0.010** | **0.003** | **0.017** | **0.0052** | **0.008** |
| 2-year cumulative TyG vs 3-year mean change TyG model | 0.040 | 0.022 | 0.058 | ＜0.001 | **＜0.001** |
| 3-year cumulative TyG vs 3-year mean change TyG model | 0.030 | 0.012 | 0.048 | ＜0.001 | **＜0.001** |

PMM, predictive mean matching; AUC, area under the curve; other abbreviations as in Table 1. ΔAUC: the difference between the two models; adjusted P-value: P values adjusted for multiple testing using Benjamini-Hochberg procedures, respectively.

**TableS15.** Pairwise comparison of cardiovascular disease prediction models based on **TyG-BMI** indices(data imputed with PMM)

| **Model Pairs** | **ΔAUC** | **Lower95%CI** | **Upper95%CI** | ***P*-value** | **Adjusted *P*-value** |
| --- | --- | --- | --- | --- | --- |
| base model vs basline TyG-BMI | -0.029 | -0.045 | -0.014 | ＜0.001 | **＜0.001** |
| base model vs 2-year cumulative TyG-BMI | -0.033 | -0.052 | -0.015 | ＜0.001 | **＜0.001** |
| base model vs 3-year cumulative TyG-BMI | -0.036 | -0.057 | -0.015 | ＜0.001 | **＜0.001** |
| base model vs 3-year mean change TyG-BMI | 0.008 | -0.006 | 0.022 | 0.281 | 0.327 |
| basline TyG-BMI vs 2-year cumulative TyG-BMI | -0.004 | -0.012 | 0.004 | 0.289 | 0.327 |
| basline TyG-BMI vs 3-year cumulative TyG-BMI | -0.007 | -0.019 | 0.005 | 0.244 | 0.321 |
| basline TyG-BMI vs 3-year mean change TyG-BMI | 0.037 | 0.014 | 0.060 | 0.002 | **0.004** |
| 2-year cumulative TyG-BMI vs 3-year cumulative TyG-BMI | -0.003 | -0.008 | 0.003 | 0.301 | 0.327 |
| 2-year cumulative TyG-BMI vs 3-year mean change TyG-BMI | 0.041 | 0.017 | 0.065 | ＜0.001 | **＜0.001** |
| 3-year cumulative TyG-BMI vs 3-year mean change TyG-BMI | 0.044 | 0.021 | 0.067 | ＜0.001 | **0.002** |

PMM, predictive mean matching; AUC, area under the curve; other abbreviations as in Table 1. ΔAUC: the difference between the two models; adjusted P-value: P values adjusted for multiple testing using Benjamini-Hochberg procedures, respectively.

**TableS16.** Pairwise comparison of cardiovascular disease prediction models based on **TyG-WC** Indices(data imputed with PMM)

| **Model Pairs** | **ΔAUC** | **Lower95%CI** | **Upper95%CI** | ***P*-value** | **Adjusted *P*-value** |
| --- | --- | --- | --- | --- | --- |
| base model vs basline TyG-WC | -0.072 | -0.104 | -0.041 | ＜0.001 | **＜0.001** |
| base model vs 2-year cumulative TyG-WC | -0.071 | -0.104 | -0.039 | ＜0.001 | **＜0.001** |
| base model vs 3-year cumulative TyG-WC | -0.067 | -0.100 | -0.034 | ＜0.001 | **＜0.001** |
| base model vs 3-year mean change TyG-WC | -0.003 | -0.012 | 0.006 | 0.484 | 0.635 |
| basline TyG-WC vs 2-year cumulative TyG-WC | 0.001 | -0.012 | 0.014 | 0.880 | 0.880 |
| basline TyG-WC vs 3-year cumulative TyG-WC | 0.006 | -0.014 | 0.026 | 0.568 | 0.702 |
| basline TyG-WC vs 3-year mean change TyG-WC | 0.070 | 0.040 | 0.099 | ＜0.001 | **＜0.001** |
| 2-year cumulative TyG-WC vs 3-year cumulative TyG-WC | 0.005 | -0.005 | 0.015 | 0.326 | 0.483 |
| 2-year cumulative TyG-WC vs 3-year mean change TyG-WC | 0.069 | 0.037 | 0.101 | ＜0.001 | **＜0.001** |
| 3-year cumulative TyG-WC vs 3-year mean change TyG-WC | 0.064 | 0.031 | 0.097 | ＜0.001 | **＜0.001** |

PMM, predictive mean matching; AUC, area under the curve; other abbreviations as in Table 1. ΔAUC: the difference between the two models; adjusted P-value: P values adjusted for multiple testing using Benjamini-Hochberg procedures, respectively.

**TableS17.** Pairwise comparison of cardiovascular disease prediction models based on **TyG-WHtR** Indices(data imputed with PMM)

| **Model Pairs** | **ΔAUC** | **Lower95%CI** | **Upper95%CI** | ***P*-value** | **Adjusted *P*-value** |
| --- | --- | --- | --- | --- | --- |
| base model vs basline TyG-WHtR | -0.061 | -0.088 | -0.035 | ＜0.001 | **＜0.001** |
| base model vs 2-year cumulative TyG-WHtR | -0.059 | -0.086 | -0.032 | ＜0.001 | **＜0.001** |
| base model vs 3-year cumulative TyG-WHtR | -0.054 | -0.08 | -0.028 | ＜0.001 | **＜0.001** |
| base model vs 3-year mean change TyG-WHtR | -0.003 | -0.012 | 0.006 | 0.409 | 0.505 |
| basline TyG-WHtR vs 2-year cumulative TyG-WHtR | 0.003 | -0.009 | 0.014 | 0.660 | 0.693 |
| basline TyG-WHtR vs 3-year cumulative TyG-WHtR | 0.007 | -0.01 | 0.024 | 0.409 | 0.505 |
| basline TyG-WHtR vs 3-year mean change TyG-WHtR | 0.058 | 0.034 | 0.082 | ＜0.001 | **＜0.001** |
| 2-year cumulative TyG-WHtR vs 3-year cumulative TyG-WHtR | 0.005 | -0.004 | 0.013 | 0.270 | 0.405 |
| 2-year cumulative TyG-WHtR vs 3-year mean change TyG-WHtR | 0.055 | 0.030 | 0.081 | ＜0.001 | **＜0.001** |
| 3-year cumulative TyG-WHtR vs 3-year mean change TyG-WHtR | 0.051 | 0.025 | 0.077 | ＜0.001 | **＜0.001** |

PMM, predictive mean matching; AUC, area under the curve; other abbreviations as in Table 1. ΔAUC: the difference between the two models; adjusted P-value: P values adjusted for multiple testing using Benjamini-Hochberg procedures, respectively.

**TableS18**. Pairwise comparison of cardiovascular disease prediction models based on **baseline** TyG-related indices(data imputed with CART)

| **Model Pairs** | **ΔAUC** | **Lower95%CI** | **Upper95%CI** | ***P*-value** | **Adjusted *P*-value** |
| --- | --- | --- | --- | --- | --- |
| base model vs basline TyG model | -0.039 | -0.056 | -0.022 | ＜0.001 | **＜0.001** |
| base model vs basline TyG-BMI model | -0.030 | -0.046 | -0.014 | ＜0.001 | **＜0.001** |
| base model vs basline TyG-WC model | -0.072 | -0.103 | -0.041 | ＜0.001 | **＜0.001** |
| base model vs basline TyG-WHtR model | -0.061 | -0.087 | -0.035 | ＜0.001 | **＜0.001** |
| basline TyG modelvs basline TyG-BMI model | 0.009 | -0.007 | 0.024 | 0.256 | 0.256 |
| basline TyG model vs basline TyG-WC model | -0.033 | -0.056 | -0.010 | 0.006 | **0.008** |
| basline TyG model vs basline TyG-WHtR model | -0.022 | -0.041 | -0.002 | 0.030 | **0.033** |
| basline TyG-BMI model vs basline TyG-WC model | -0.042 | -0.065 | -0.019 | ＜0.001 | **＜0.001** |
| basline TyG-BMI model vs basline TyG-WHtR model | -0.031 | -0.048 | -0.013 | ＜0.001 | **0.001** |
| basline TyG-WC model vs basline TyG-WHtR model | 0.011 | 0.002 | 0.021 | 0.021 | **0.026** |

CART,classification and regression trees; AUC, area under the curve; other abbreviations as in Table 1. ΔAUC: the difference between the two models; adjusted P-value: P values adjusted for multiple testing using Benjamini-Hochberg procedures, respectively.

**TableS19.** Pairwise comparison of cardiovascular disease prediction models based on **2-year cumulative** TyG-related indices(data imputed with CART)

| **Model Pairs** | **ΔAUC** | **Lower95%CI** | **Upper95%CI** | ***P*-value** | **Adjusted *P*-value** |
| --- | --- | --- | --- | --- | --- |
| base model vs 2-year cumulative TyG model | -0.048 | -0.069 | -0.027 | ＜0.001 | **＜0.001** |
| base model vs 2-year cumulative TyG-BMI model | -0.034 | -0.055 | -0.013 | 0.001 | **0.003** |
| base model vs 2-year cumulative TyG-WC model | -0.070 | -0.103 | -0.037 | ＜0.001 | **＜0.001** |
| base model vs 2-year cumulative TyG-WHtR model | -0.057 | -0.086 | -0.028 | ＜0.001 | **＜0.001** |
| 2-year cumulative TyG model vs 2-year cumulative TyG-BMI model | 0.014 | -0.004 | 0.033 | 0.134 | 0.149 |
| 2-year cumulative TyG model vs 2-year cumulative TyG-WC model | -0.022 | -0.047 | 0.003 | 0.086 | 0.108 |
| 2-year cumulative TyG model vs 2-year cumulative TyG-WHtR model | -0.009 | -0.030 | 0.012 | 0.420 | 0.420 |
| 2-year cumulative TyG-BMI model vs 2-year cumulative TyG-WC model | -0.036 | -0.058 | -0.014 | 0.002 | **0.003** |
| 2-year cumulative TyG-BMI model vs 2-year cumulative TyG-WHtR model | -0.023 | -0.040 | -0.006 | 0.010 | **0.017** |
| 2-year cumulative TyG-WC model vs 2-year cumulative TyG-WHtR model | 0.013 | 0.002 | 0.024 | 0.018 | **0.026** |

CART,classification and regression trees; AUC, area under the curve; other abbreviations as in Table 1. ΔAUC: the difference between the two models; adjusted P-value: P values adjusted for multiple testing using Benjamini-Hochberg procedures, respectively.

**TableS20.** Pairwise comparison of cardiovascular disease prediction models based on **3-year cumulative** TyG-related indices(data imputed with CART)

| **Model Pairs** | **ΔAUC** | **Lower95%CI** | **Upper95%CI** | ***P*-value** | **Adjusted *P*-value** |
| --- | --- | --- | --- | --- | --- |
| base model vs 3-year cumulative TyG model | -0.039 | -0.057 | -0.020 | ＜0.001 | **＜0.001** |
| base model vs 3-year cumulative TyG-BMI model | -0.037 | -0.060 | -0.014 | 0.001 | **0.004** |
| base model vs 3-year cumulative TyG-WC model | -0.066 | -0.098 | -0.034 | ＜0.001 | **＜0.001** |
| base model vs 3-year cumulative TyG-WHtR model | -0.052 | -0.080 | -0.025 | ＜0.001 | **＜0.001** |
| 3-year cumulative TyG model vs 3-year cumulative TyG-BMI model | 0.002 | -0.018 | 0.022 | 0.872 | 0.872 |
| 3-year cumulative TyG model vs 3-year cumulative TyG-WC model | -0.027 | -0.051 | -0.003 | 0.025 | **0.036** |
| 3-year cumulative TyG model vs 3-year cumulative TyG-WHtR model | -0.014 | -0.034 | 0.007 | 0.186 | 0.207 |
| 3-year cumulative TyG-BMI model vs 3-year cumulative TyG-WC model | -0.029 | -0.048 | -0.009 | 0.004 | **0.008** |
| 3-year cumulative TyG-BMI model vs 3-year cumulative TyG-WHtR model | -0.015 | -0.030 | -0.001 | 0.042 | 0.053 |
| 3-year cumulative TyG-WC model vs 3-year cumulative TyG-WHtR model | 0.013 | 0.002 | 0.025 | 0.025 | **0.036** |

CART,classification and regression trees; AUC, area under the curve; other abbreviations as in Table 1. ΔAUC: the difference between the two models; adjusted P-value: P values adjusted for multiple testing using Benjamini-Hochberg procedures, respectively.

**TableS21.** Pairwise comparison of cardiovascular disease prediction models based on **3-year mean change** TyG-related indices(data imputed with CART)

| **Model Pairs** | **ΔAUC** | **Lower95%CI** | **Upper95%CI** | ***P*-value** | **Adjusted *P*-value** |
| --- | --- | --- | --- | --- | --- |
| base model vs 3-year mean change TyG | -0.008 | -0.024 | 0.008 | 0.282 | 0.437 |
| base model vs 3-year mean change TyG-BMI | 0.006 | -0.006 | 0.017 | 0.322 | 0.437 |
| base model vs 3-year mean change TyG-WC | -0.004 | -0.009 | 0.001 | 0.122 | 0.350 |
| base model vs 3-year mean change TyG-WHtR | -0.004 | -0.010 | 0.001 | 0.111 | 0.350 |
| 3-year mean change TyG vs 3-year mean change TyG-BMI | 0.013 | -0.004 | 0.031 | 0.127 | 0.350 |
| 3-year mean change TyG vs 3-year mean change TyG-WC | 0.004 | -0.011 | 0.018 | 0.554 | 0.592 |
| 3-year mean change TyG vs 3-year mean change TyG-WHtR | 0.003 | -0.011 | 0.018 | 0.592 | 0.592 |
| 3-year mean change TyG-BMI vs 3-year mean change TyG-WC | -0.010 | -0.024 | 0.004 | 0.175 | 0.350 |
| 3-year mean change TyG-BMI vs 3-year mean change TyG-WHtR | -0.010 | -0.025 | 0.004 | 0.168 | 0.350 |
| 3-year mean change TyG-WC vs 3-year mean change TyG-WHtR | 0.000 | -0.001 | 0.000 | 0.349 | 0.437 |

CART,classification and regression trees; AUC, area under the curve; other abbreviations as in Table 1. ΔAUC: the difference between the two models; adjusted P-value: P values adjusted for multiple testing using Benjamini-Hochberg procedures, respectively.

**TableS22.** Pairwise comparison of cardiovascular disease prediction models based on **TyG** indices(data imputed with CART)

| **Model Pairs** | **ΔAUC** | **Lower95%CI** | **Upper95%CI** | ***P*-value** | **Adjusted *P*-value** |
| --- | --- | --- | --- | --- | --- |
| base model vs basline TyG model | -0.039 | -0.057 | -0.021 | ＜0.001 | **＜0.001** |
| base model vs 2-year cumulative TyG model | -0.048 | -0.069 | -0.027 | ＜0.001 | **＜0.001** |
| base model vs 3-year cumulative TyG model | -0.039 | -0.057 | -0.020 | ＜0.001 | **＜0.001** |
| base model vs 3-year mean change TyG model | -0.008 | -0.024 | 0.008 | 0.283 | 0.313 |
| basline TyG vs 2-year cumulative TyG model | -0.009 | -0.019 | 0.001 | 0.078 | 0.102 |
| basline TyG vs 3-year cumulative TyG model | 0.000 | -0.014 | 0.015 | 0.947 | 0.947 |
| basline TyG vs 3-year mean change TyG model | 0.032 | 0.018 | 0.045 | ＜0.001 | **＜0.001** |
| 2-year cumulative TyG vs 3-year cumulative TyG model | 0.009 | 0.001 | 0.018 | 0.028 | **0.041** |
| 2-year cumulative TyG vs 3-year mean change TyG model | 0.040 | 0.022 | 0.059 | ＜0.001 | **＜0.001** |
| 3-year cumulative TyG vs 3-year mean change TyG model | 0.031 | 0.010 | 0.052 | 0.004 | **0.008** |

CART,classification and regression trees; AUC, area under the curve; other abbreviations as in Table 1. ΔAUC: the difference between the two models; adjusted P-value: P values adjusted for multiple testing using Benjamini-Hochberg procedures, respectively.

**TableS23.** Pairwise comparison of cardiovascular disease prediction models based on **TyG-BMI** indices(data imputed with CART)

| **Model Pairs** | **ΔAUC** | **Lower95%CI** | **Upper95%CI** | ***P*-value** | **Adjusted *P*-value** |
| --- | --- | --- | --- | --- | --- |
| base model vs basline TyG-BMI | -0.030 | -0.046 | -0.014 | ＜0.001 | **0.002** |
| base model vs 2-year cumulative TyG-BMI | -0.034 | -0.054 | -0.014 | ＜0.001 | **0.003** |
| base model vs 3-year cumulative TyG-BMI | -0.037 | -0.059 | -0.015 | ＜0.001 | **0.002** |
| base model vs 3-year mean change TyG-BMI | 0.006 | -0.006 | 0.018 | 0.342 | 0.399 |
| basline TyG-BMI vs 2-year cumulative TyG-BMI | -0.004 | -0.012 | 0.004 | 0.374 | 0.413 |
| basline TyG-BMI vs 3-year cumulative TyG-BMI | -0.007 | -0.019 | 0.005 | 0.267 | 0.330 |
| basline TyG-BMI vs 3-year mean change TyG-BMI | 0.036 | 0.014 | 0.059 | 0.002 | **0.003** |
| 2-year cumulative TyG-BMI vs 3-year cumulative TyG-BMI | -0.003 | -0.009 | 0.002 | 0.252 | 0.330 |
| 2-year cumulative TyG-BMI vs 3-year mean change TyG-BMI | 0.04 | 0.016 | 0.063 | 0.001 | **0.003** |
| 3-year cumulative TyG-BMI vs 3-year mean change TyG-BMI | 0.043 | 0.019 | 0.066 | ＜0.001 | **0.002** |

CART,classification and regression trees; AUC, area under the curve; other abbreviations as in Table 1. ΔAUC: the difference between the two models; adjusted P-value: P values adjusted for multiple testing using Benjamini-Hochberg procedures, respectively.

**TableS24.** Pairwise comparison of cardiovascular disease prediction models based on **TyG-WC** indices(data imputed with CART)

| Model Pairs | ΔAUC | Lower95%CI | Upper95%CI | *P*-value | Adjusted *P*-value |
| --- | --- | --- | --- | --- | --- |
| base model vs basline TyG-WC | -0.072 | -0.103 | -0.041 | ＜0.001 | ＜0.001 |
| base model vs 2-year cumulative TyG-WC | -0.07 | -0.103 | -0.037 | ＜0.001 | ＜0.001 |
| base model vs 3-year cumulative TyG-WC | -0.066 | -0.099 | -0.032 | ＜0.001 | ＜0.001 |
| base model vs 3-year mean change TyG-WC | -0.004 | -0.009 | 0.001 | 0.116 | 0.187 |
| basline TyG-WC vs 2-year cumulative TyG-WC | 0.002 | -0.011 | 0.016 | 0.734 | 0.771 |
| basline TyG-WC vs 3-year cumulative TyG-WC | 0.006 | -0.014 | 0.026 | 0.524 | 0.647 |
| basline TyG-WC vs 3-year mean change TyG-WC | 0.068 | 0.039 | 0.097 | ＜0.001 | **＜0.001** |
| 2-year cumulative TyG-WC vs 3-year cumulative TyG-WC | 0.004 | -0.005 | 0.014 | 0.387 | 0.508 |
| 2-year cumulative TyG-WC vs 3-year mean change TyG-WC | 0.066 | 0.034 | 0.098 | ＜0.001 | **＜0.001** |
| 3-year cumulative TyG-WC vs 3-year mean change TyG-WC | 0.062 | 0.028 | 0.095 | ＜0.001 | **＜0.001** |

CART,classification and regression trees; AUC, area under the curve; other abbreviations as in Table 1. ΔAUC: the difference between the two models; adjusted P-value: P values adjusted for multiple testing using Benjamini-Hochberg procedures, respectively.

**TableS25.** Pairwise comparison of cardiovascular disease prediction models based on **TyG-WHtR** indices(data imputed with CART)

| **Model Pairs** | **ΔAUC** | **Lower95%CI** | **Upper95%CI** | ***P*-value** | **Adjusted *P*-value** |
| --- | --- | --- | --- | --- | --- |
| base model vs basline TyG-WHtR | -0.061 | -0.087 | -0.035 | ＜0.001 | **＜0.001** |
| base model vs 2-year cumulative TyG-WHtR | -0.057 | -0.084 | -0.030 | ＜0.001 | **＜0.001** |
| base model vs 3-year cumulative TyG-WHtR | -0.052 | -0.079 | -0.026 | ＜0.001 | **＜0.001** |
| base model vs 3-year mean change TyG-WHtR | -0.004 | -0.01 | 0.001 | 0.115 | 0.186 |
| basline TyG-WHtR vs 2-year cumulative TyG-WHtR | 0.004 | -0.008 | 0.016 | 0.486 | 0.537 |
| basline TyG-WHtR vs 3-year cumulative TyG-WHtR | 0.008 | -0.008 | 0.025 | 0.318 | 0.401 |
| basline TyG-WHtR vs 3-year mean change TyG-WHtR | 0.057 | 0.033 | 0.080 | ＜0.001 | **＜0.001** |
| 2-year cumulative TyG-WHtR vs 3-year cumulative TyG-WHtR | 0.004 | -0.004 | 0.013 | 0.310 | 0.401 |
| 2-year cumulative TyG-WHtR vs 3-year mean change TyG-WHtR | 0.052 | 0.027 | 0.078 | ＜0.001 | **＜0.001** |
| 3-year cumulative TyG-WHtR vs 3-year mean change TyG-WHtR | 0.048 | 0.022 | 0.075 | ＜0.001 | **＜0.001** |

CART,classification and regression trees; AUC, area under the curve; other abbreviations as in Table 1. ΔAUC: the difference between the two models; adjusted P-value: P values adjusted for multiple testing using Benjamini-Hochberg procedures, respectively.

**TableS26.** Comparison results of AUCs across time points for different models (data imputed with PMM)

| **Model** | **Comparison** | **ΔAUC 95%CI** | ***P*-value** | **Adjusted *P*-value** |
| --- | --- | --- | --- | --- |
| base model | 48vs60 | -0.014(-0.069-0.042) | 0.632 | 0.632 |
|  | 48vs72 | 0.014 (-0.043-0.071) | 0.632 | 0.632 |
|  | 60vs72 | 0.028 (-0.029-0.084) | 0.342 | 0.632 |
| Baseline TyG | 48vs60 | -0.015(-0.057-0.027) | 0.490 | 0.535 |
|  | 48vs72 | -0.029(-0.076-0.018) | 0.221 | 0.535 |
|  | 60vs72 | -0.014(-0.060-0.031) | 0.535 | 0.535 |
| Baseline TyG-BMI | 48vs60 | -0.015(-0.060-0.029) | 0.499 | 0.748 |
|  | 48vs72 | -0.019(-0.066-0.027) | 0.415 | 0.748 |
|  | 60vs72 | -0.004(-0.050-0.042) | 0.865 | 0.865 |
| Baseline TyG-WC | 48vs60 | -0.011(-0.065-0.044) | 0.702 | 0.702 |
|  | 48vs72 | -0.054(-0.109-0.001) | 0.055 | 0.165 |
|  | 60vs72 | -0.043(-0.100-0.013) | 0.134 | 0.202 |
| Baseline TyG-WHtR | 48vs60 | -0.011(-0.063-0.041) | 0.678 | 0.678 |
|  | 48vs72 | -0.046(-0.099-0.006) | 0.085 | 0.253 |
|  | 60vs72 | -0.035(-0.088-0.018) | 0.193 | 0.289 |
| 2-year cumulative TyG | 48vs60 | -0.014(-0.061-0.032) | 0.553 | 0.717 |
|  | 48vs72 | -0.036(-0.085-0.012) | 0.144 | 0.432 |
|  | 60vs72 | -0.022(-0.070-0.025) | 0.359 | 0.538 |
| 2-year cumulative TyG-BMI | 48vs60 | -0.015(-0.060-0.029) | 0.495 | 0.742 |
|  | 48vs72 | -0.022(-0.068-0.023) | 0.329 | 0.742 |
|  | 60vs72 | -0.007(-0.051-0.037) | 0.754 | 0.754 |
| 2-year cumulative TyG-WC | 48vs60 | -0.011(-0.065-0.043) | 0.695 | 0.695 |
|  | 48vs72 | -0.051(-0.103-0.002) | 0.059 | 0.178 |
|  | 60vs72 | -0.040(-0.094-0.014) | 0.149 | 0.224 |
| 2-year cumulative TyG-WHtR | 48vs60 | -0.012(-0.062-0.039) | 0.654 | 0.654 |
|  | 48vs72 | -0.043(-0.091-0.006) | 0.086 | 0.258 |
|  | 60vs72 | -0.031(-0.080-0.017) | 0.209 | 0.313 |
| 3-year cumulative TyG | 48vs60 | -0.014(-0.061-0.032) | 0.541 | 0.625 |
|  | 48vs72 | -0.026(-0.072-0.020) | 0.273 | 0.625 |
|  | 60vs72 | -0.011(-0.058-0.035) | 0.625 | 0.625 |
| 3-year cumulative TyG-BMI | 48vs60 | -0.016(-0.063-0.030) | 0.488 | 0.732 |
|  | 48vs72 | -0.025(-0.069-0.020) | 0.281 | 0.732 |
|  | 60vs72 | -0.008(-0.056-0.039) | 0.733 | 0.733 |
| 3-year cumulative TyG-WC | 48vs60 | -0.013(-0.062-0.037) | 0.611 | 0.611 |
|  | 48vs72 | -0.045(-0.095-0.005) | 0.075 | 0.225 |
|  | 60vs72 | -0.033(-0.083-0.018) | 0.207 | 0.311 |
| 3-year cumulative TyG-WHtR | 48vs60 | -0.013(-0.057-0.031) | 0.562 | 0.562 |
|  | 48vs72 | -0.038(-0.085-0.009) | 0.114 | 0.342 |
|  | 60vs72 | -0.025(-0.072-0.022) | 0.303 | 0.455 |
| 3-year mean change TyG | 48vs60 | -0.013(-0.063-0.037) | 0.598 | 0.815 |
|  | 48vs72 | 0.006 (-0.045-0.058) | 0.815 | 0.815 |
|  | 60vs72 | 0.020 (-0.031-0.070) | 0.446 | 0.815 |
| 3-year mean change TyG-BMI | 48vs60 | -0.015(-0.079-0.050) | 0.656 | 0.656 |
|  | 48vs72 | 0.026 (-0.036-0.088) | 0.417 | 0.626 |
|  | 60vs72 | 0.040 (-0.023-0.104) | 0.213 | 0.626 |
| 3-year mean change TyG-WC | 48vs60 | -0.013(-0.067-0.041) | 0.630 | 0.688 |
|  | 48vs72 | 0.011 (-0.043-0.065) | 0.688 | 0.688 |
|  | 60vs72 | 0.024 (-0.029-0.077) | 0.370 | 0.688 |
| 3-year mean change TyG-WHtR | 48vs60 | -0.013(-0.068-0.041) | 0.635 | 0.701 |
|  | 48vs72 | 0.011 (-0.043-0.064) | 0.701 | 0.701 |
|  | 60vs72 | 0.024 (-0.031-0.078) | 0.391 | 0.701 |

AUC, area under the curve; other abbreviations as in Table 1. ΔAUC: the difference between the two models; adjusted P-value: P values adjusted for multiple testing using Benjamini-Hochberg procedures, respectively.

**TableS27.** Comparison results of AUCs across time points for different models (data imputed with CART)

| **Model** | **Comparison** | **ΔAUC 95%CI** | ***P*-value** | **Adjusted *P*-value** |
| --- | --- | --- | --- | --- |
| base model | 48vs60 | -0.014 (-0.071-0.043) | 0.637 | 0.651 |
|  | 48vs72 | 0.013 (-0.044-0.071) | 0.651 | 0.651 |
|  | 60vs72 | 0.027 (-0.030-0.084) | 0.351 | 0.651 |
| Baseline TyG | 48vs60 | -0.015 (-0.060-0.031) | 0.527 | 0.527 |
|  | 48vs72 | -0.030 (-0.077-0.017) | 0.207 | 0.527 |
|  | 60vs72 | -0.016 (-0.063-0.032) | 0.517 | 0.527 |
| Baseline TyG-BMI | 48vs60 | -0.016 (-0.061-0.029) | 0.473 | 0.710 |
|  | 48vs72 | -0.021 (-0.068-0.025) | 0.368 | 0.710 |
|  | 60vs72 | -0.005 (-0.052-0.042) | 0.839 | 0.839 |
| Baseline TyG-WC | 48vs60 | -0.010 (-0.067-0.046) | 0.724 | 0.724 |
|  | 48vs72 | -0.054 (-0.111-0.002) | 0.058 | 0.175 |
|  | 60vs72 | -0.044 (-0.099-0.011) | 0.117 | 0.176 |
| Baseline TyG-WHtR | 48vs60 | -0.011 (-0.062-0.040) | 0.677 | 0.677 |
|  | 48vs72 | -0.047 (-0.101-0.007) | 0.088 | 0.265 |
|  | 60vs72 | -0.036 (-0.090-0.018) | 0.190 | 0.285 |
| 2-year cumulative TyG | 48vs60 | -0.014 (-0.062-0.034) | 0.574 | 0.574 |
|  | 48vs72 | -0.037 (-0.084-0.010) | 0.119 | 0.356 |
|  | 60vs72 | -0.023 (-0.069-0.022) | 0.312 | 0.468 |
| 2-year cumulative TyG-BMI | 48vs60 | -0.016 (-0.061-0.030) | 0.497 | 0.745 |
|  | 48vs72 | -0.023 (-0.071-0.025) | 0.351 | 0.745 |
|  | 60vs72 | -0.007 (-0.054-0.040) | 0.770 | 0.770 |
| 2-year cumulative TyG-WC | 48vs60 | -0.010 (-0.064-0.044) | 0.716 | 0.716 |
|  | 48vs72 | -0.049 (-0.101-0.002) | 0.060 | 0.181 |
|  | 60vs72 | -0.039 (-0.092-0.013) | 0.142 | 0.213 |
| 2-year cumulative TyG-WHtR | 48vs60 | -0.011 (-0.059-0.037) | 0.653 | 0.653 |
|  | 48vs72 | -0.041 (-0.091-0.009) | 0.109 | 0.326 |
|  | 60vs72 | -0.030 (-0.080-0.019) | 0.232 | 0.347 |
| 3-year cumulative TyG | 48vs60 | -0.014 (-0.059-0.030) | 0.528 | 0.570 |
|  | 48vs72 | -0.028 (-0.073-0.018) | 0.234 | 0.570 |
|  | 60vs72 | -0.013 (-0.059-0.032) | 0.570 | 0.570 |
| 3-year cumulative TyG-BMI | 48vs60 | -0.016 (-0.061-0.028) | 0.467 | 0.701 |
|  | 48vs72 | -0.025 (-0.070-0.020) | 0.278 | 0.701 |
|  | 60vs72 | -0.008 (-0.054-0.037) | 0.711 | 0.711 |
| 3-year cumulative TyG-WC | 48vs60 | -0.012 (-0.062-0.037) | 0.630 | 0.630 |
|  | 48vs72 | -0.045 (-0.094-0.004) | 0.074 | 0.222 |
|  | 60vs72 | -0.033 (-0.084-0.019) | 0.211 | 0.316 |
| 3-year cumulative TyG-WHtR | 48vs60 | -0.013 (-0.060-0.035) | 0.603 | 0.603 |
|  | 48vs72 | -0.037 (-0.083-0.010) | 0.121 | 0.363 |
|  | 60vs72 | -0.024 (-0.073-0.024) | 0.326 | 0.489 |
| 3-year mean change TyG | 48vs60 | -0.013 (-0.064-0.037) | 0.605 | 0.841 |
|  | 48vs72 | 0.005 (-0.046-0.056) | 0.841 | 0.841 |
|  | 60vs72 | 0.019 (-0.030-0.067) | 0.454 | 0.841 |
| 3-year mean change TyG-BMI | 48vs60 | -0.014 (-0.074-0.046) | 0.656 | 0.656 |
|  | 48vs72 | 0.023 (-0.037-0.083) | 0.451 | 0.656 |
|  | 60vs72 | 0.037 (-0.024-0.098) | 0.240 | 0.656 |
| 3-year mean change TyG-WC | 48vs60 | -0.013 (-0.064-0.038) | 0.617 | 0.733 |
|  | 48vs72 | 0.009 (-0.044-0.062) | 0.733 | 0.733 |
|  | 60vs72 | 0.022 (-0.033-0.077) | 0.429 | 0.733 |
| 3-year mean change TyG-WHtR | 48vs60 | -0.013 (-0.064-0.038) | 0.616 | 0.746 |
|  | 48vs72 | 0.009 (-0.045-0.062) | 0.746 | 0.746 |
|  | 60vs72 | 0.022 (-0.031-0.075) | 0.420 | 0.746 |

CART,classification and regression trees; AUC, area under the curve; other abbreviations as in Table 1. ΔAUC: the difference between the two models; adjusted P-value: P values adjusted for multiple testing using Benjamini-Hochberg procedures, respectively.

**Fig.S1** Kaplan-Meier curves for cardiovascular events cumulative incidence by CKM syndrome stages 0–3(data imputed with CART).

CART, classification and regression trees; other abbreviations as in Table 1. Stage 0-1(blue); Stage 2 (green); Stage 3 (purple-red); Log-rank test: *P*=0.004.

**Fig.S2** Kaplan-Meier curves for cardiovascular events cumulative incidence by CKM syndrome stages 0–3(original non-imputed data).

Abbreviations as in Table 1.Stage 0-1(blue); Stage 2 (orange); Stage 3 (green). Log-rank test: *P*=0.070.

**Fig.S3** RCS analyses between baseline TyG-related indices and cardiovascular events in populations with CKM syndrome stages 0–3(data imputed with CART).

RCS, restricted cubic splines; CART,classification and regression trees; other abbreviations as in Table 1.

**Fig.S4** RCS analyses between baseline TyG-related indices and cardiovascular events in populations with CKM syndrome stages 0–3(original non-imputed data).

RCS, restricted cubic splines; other abbreviations as in Table 1.

**Fig.S5** Predictive performance of TyG-related indices for 4-year and 5-year cardiovascular events in populations with CKM syndrome stages 0–3(data imputed with PMM).

PMM, predictive mean matching; AUC, area under the curve; PMM, predictive mean matching; other abbreviations as in Table 1.

**Fig.S6** Predictive performance of TyG-related indices for cardiovascular events in populations with CKM syndrome stages 0–3(data imputed with CART)

CART,classification and regression trees; AUC, area under the curve; CART,classification and regression trees; other abbreviations as in Table 1.

**Fig.S7** Predictive performance of TyG-related indices for 4-year and 5-year cardiovascular events in populations with CKM syndrome stages 0–3(data imputed with CART).

CART,classification and regression trees; AUC, area under the curve;CART,classification and regression trees; other abbreviations as in Table 1.

**Fig.S8** Longitudinal trajectories of TyG-related indices in populations with CKM syndrome stages 0–3 stratified by CVD status(data imputed with CART).

CART,classification and regression trees; other abbreviations as in Table 1; data are presented as pooled mean values with standard error (SE) bars. The P-values represent the interaction between time and CVD status.

**Reference**

[1]Ndumele CE, Rangaswami J, Chow SL, Neeland IJ, Tuttle KR, Khan SS, et al. Cardiovascular-kidney-metabolic health: A presidential advisory from the American heart association. Circulation. American Heart Association; 2023;148:1606–35. doi.org/10.1161/CIR.0000000000001184
[2]Khan SS, Coresh J, Pencina MJ, Ndumele CE, Rangaswami J, Chow SL, et al. Novel prediction equations for absolute risk assessment of total cardiovascular disease incorporating cardiovascular-kidney-metabolic health: a scientific statement from the American Heart Association. Circulation 2023;148(24):1982–2004.doi: 10.1161/cir.0000000000001191
